# Assessment of Oxidative Stress, Vitellogenin, and Human Health Risks Derived From Nile Tilapia Exposed to Metals and Metalloids in Southeastern Brazil

**DOI:** 10.1002/tox.70094

**Published:** 2026-04-15

**Authors:** Julia Araújo Alves, Priscila M. O. Muniz Cunha, Shênia Patrícia Corrêa Novo, Lorena Oliveira Souza Soares, Tatiana Dillenburg Saint‐Pierre, Fábio Veríssimo Correia, André Luís de Sá Salomão, Rachel Ann Hauser‐Davis, Enrico Mendes Saggioro

**Affiliations:** ^1^ Environmental Health Assessment and Promotion Laboratory (LAPSA) Oswaldo Cruz Institute (IOC), Oswaldo Cruz Foundation (Fiocruz) Rio de Janeiro Brazil; ^2^ Bioremediation and Phytotechnology Laboratory (LABIFI), Department of Environmental Engineering Rio de Janeiro State University (UERJ) Rio de Janeiro Brazil; ^3^ Laboratory of Comparative and Environmental Virology (LVCA) Oswaldo Cruz Institute (IOC), Oswaldo Cruz Foundation (Fiocruz) Rio de Janeiro Brazil; ^4^ Laboratório de Espectrometria Atômica (Labspectro), Departamento de Química Pontifícia Universidade Católica Do Rio de Janeiro (PUC‐Rio) Rio de Janeiro Brazil; ^5^ Environmental Health Laboratory, Department of Natural Sciences Federal University of the State of Rio de Janeiro (UNIRIO) Rio de Janeiro Brazil

**Keywords:** fish, organosomatic indices, subcellular metals and metalloids, urban aquatic pollution, vitellogenesis

## Abstract

Urban coastal lagoons are highly vulnerable ecosystems increasingly affected by metal contamination, as well as multiple anthropogenic stressors. This study evaluated ecotoxicological and human health risks associated with chronic metal exposure in Nile tilapia ( *Oreochromis niloticus* ) from an anthropogenically impacted lagoon in southeastern Brazil. Monitoring was conducted over one year (2022–2023) across four seasonal campaigns. Fifty‐seven tilapia specimens were collected during dry and rainy seasons, and liver and muscle tissue were analyzed for antioxidant biomarkers (superoxide dismutase–SOD, glutathione S‐transferase–GST, reduced glutathione–GSH, total antioxidant capacity–TAC), oxidative damage (lipid peroxidation–LPO, protein carbonyl–PTC), and metal detoxification (metallothionein–MT). Endocrine disruption was assessed via plasma vitellogenin (VTG), and human health risks were estimated. Metal and metalloid diversity was higher in the dry season (12 vs. 7 elements), with some sites (P1–P3) presenting critical levels. Metals and metalloids in MT‐enriched subcellular tissue fractions revealed seasonal shifts in As, Co, Rb, and Se, with Cd, Hg, and Pb detected only in liver during the dry period. Sex differences observed regarding GSI (females > males; *p* ≤ 0.05) aligned with higher male VTG levels. Antioxidant responses were higher in the dry season (hepatic SOD +81.9%), while oxidative damage increased substantially in both tissues (muscle +75.7%, liver +79.6%). The applied integrated biomarker–metal approach revealed a clear dry‐season intensification of metal and metalloid exposure and biological responses, emphasizing endocrine and oxidative alterations and highlighting the need for seasonal biomonitoring efforts.

## 1 Introduction

Human society is intrinsically associated to coastal ecosystems, which play a vital role in ecological stability and the provision of essential services [1]. These ecosystems, however, are increasingly threatened by multiple anthropogenic stressors, including overfishing [2], the presence of invasive species [3, 4], disease outbreaks [5], population growth [6], climate change [7], and the intense exploitation of coastal zones for recreation and tourism [8, 9]. Among these threats, chemical pollution from urban and industrial sources stands out as a critical and widespread ecosystem degradation driver [10, 11]. Metals and metalloids are of particular concern in this sense, due to their persistence, ability to bioaccumulate and potentially toxic ecological and human health effects.

Fish are recognized as key environmental health indicators due to their broad geographic distribution, ecological relevance, and ability to bioaccumulate metals, among other contaminants (FAO [2]). Metal and metalloid pollution in fish has been associated with metabolic disorders [12], oxidative stress [13], and genetic damage [14, 15], leading to impaired reproductive function, altered growth, behavioral changes, and increased susceptibility to disease [16, 17]. Metal and metalloid contamination also poses human‐health risks through the consumption of contaminated seafood [18, 19, 20].

Metals and metalloids enter fish via gills, skin, or dietary intake [21] and are subsequently distributed among tissues and intracellular compartments [22]. This compartmentalization is key in modulating toxicity, as only part of the accumulated pool (namely, the bioavailable fraction) interacts with sensitive cellular targets [23]. However, despite extensive evidence on metal‐induced oxidative stress, little is known about how metal–biomarker interactions operate in highly urbanized coastal lagoons, where hydrodynamics, salinity gradients, and episodic contaminant pulses differ markedly from riverine or estuarine systems. These transitional waters also remain understudied in terms of how intracellular metal partitioning may predict oxidative, detoxification, or endocrine responses under chronic exposure [24].

The labile intracellular metal and metalloid fraction is considered the most toxic, as it may catalyze redox reactions and interfere with enzymatic function, leading to reactive oxygen species (ROS) overproduction andredox imbalances [25, 26], potentially causing severe damage to proteins, lipids and DNA [26]. Consequently, assessing both total metal and metalloid burdens and those associated to metallothionein (MT), a detoxification protein, provides a more accurate understanding of the toxic potential of metals and metalloids [22, 27]. The predictive value of metals in MT‐enriched subcellular fractions for explaining biomarker responses in wild fish, however, is still poorly studied, as most field studies rely solely on total metal and metalloid tissue burdens. Thus, by explicitly assessing the MT‐enriched fraction in different fish tissues, the present study addresses a major gap in evaluating how intracellular metal pools are associated to biomarker patterns in contaminated coastal lagoons.

To mitigate toxic metal and metalloid effects, fish rely on antioxidant defenses, including low‐molecular‐weight compounds like reduced glutathione (GSH) and proteins such as MT, as well as enzymes like superoxide dismutase (SOD), catalase (CAT), glutathione peroxidase (GPx), and glutathione‐S‐transferase (GST), which play essential roles in detoxification and metal complexation [28, 29]. Among these, SOD and GST were selected in the present study due to their central roles in antioxidant defense and phase II detoxification pathway participations under metal exposure scenarios. In this sense, SOD catalyzes the dismutation of superoxide radicals, constituting a primary antioxidant barrier against ROS [30], whereas GST participates in xenobiotic conjugation and cellular detoxification processes [31]. These enzymes are widely applied in ecotoxicological research as sensitive oxidative stress biomarkers in metal‐exposed fish [32, 33, 34].

Metal exposure has also been associated with endocrine disruption in several taxa [35]. Vitellogenin (VTG), an estrogen‐regulated precursor protein synthesized in females [36], is a sensitive biomarker of endocrine disruption when induced in males [37, 38]. High VTG levels in males are indicative of feminization processes driven by either natural or synthetic estrogens or other endocrine‐disrupting chemicals (EDCs), including elements presenting estrogenic activity [35, 36, 39]. Certain metals and metalloids such as As, Cd, Hg, Ni, and Zn, for example, have been shown to induce VTG production in male fish, suggesting potential estrogenic activity [36, 37].

Nile tilapia ( *Oreochromis niloticus* ) are frequently employed in ecotoxicological studies to assess metal contamination due to their global economic relevance, high consumption rates by humans, and documented accumulation of metals and metalloids in urban and industrial settings [14, 40, 41, 42]. Tilapia also serve as an ecologically and socioeconomically relevant sentinel species for lagoon systems [43], as their limited migratory behavior, high site fidelity, and continuous year‐round reproduction allow them to integrate local contaminant exposure over time [19, 44, 45]. Their omnivorous, benthic‐associated also feeding exposes them to both dissolved and sediment‐bound metals and metalloids, making them suitable bioindicators of metal dynamics in shallow, low‐renewal lagoons worldwide [43]. In metal‐contaminated lagoons, multiple biomarkers in this species have been correlated with varying metal speciation and water conditions, indicating integrated physiological responses to environmental stressors [24]. However, studies integrating metals and MT‐enriched subcellular partitioning processes with oxidative and endocrine responses in tilapia remain scarce, limiting the mechanistic understanding of chronic exposure effects.

To address these knowledge gaps, this study aimed to investigate physiological and biochemical Nile tilapia responses in a highly urbanized and metal‐contaminated lagoon in southeastern Brazil, also carrying out a human health risk assessment. It was hypothesized that resident fish would exhibit clear oxidative, detoxification, and endocrine alterations associated with chronic and seasonally modulated pollutant exposure, despite ongoing mitigation measures. The studied lagoon, thus, represents a relevant model for evaluating early biological responses and ecological risks, extensible to other urban coastal ecosystems worldwide undergoing simultaneous anthropogenic pressure and restoration interventions.

## 2 Material and Methods

### 2.1 Study Area

Piratininga Lagoon (PIR) is a shallow coastal lagoon located in the oceanic area of the municipality of Niterói, in southeastern Brazil, and forms part of the Piratininga–Itaipu Lagoon System (22°56′46.23″ S, 43°04′29.57″ W). This lagoon encompasses about 2.87 km^2^ and is characterized by a very low average depth (mean: 0.5 m), low hydrodynamics, and significant urban encroachment [46]. Water exchanges with adjacent systems occur primarily through the Camboatá Channel, an artificial connection established in 1946 linking PIR to Itaipu Lagoon [47]. Despite episodic marine influence, Piratininga Lagoon behaves predominantly as a semi‐enclosed system, with restricted renewal rates and sustained inputs from surrounding urbanized areas [48].

This lagoon was selected due to its ecological vulnerability, intense and unregulated surrounding urbanization, as well as a site of ongoing human use and municipal restoration efforts. Although the lagoon receives continuous untreated sewage and urban runoff inputs, leading to organic enrichment and contaminant accumulation [49], it also supports recreational activities and small‐scale fisheries, raising environmental and human health risk concerns.

To capture spatial heterogeneity within the lagoon, five georeferenced sampling sites (P1–P5) were established, representing distinct hydrodynamic conditions and degrees of anthropogenic influence, based on environmental criteria established in the Piratininga–Itaipu Lagoon Monitoring Plan developed in the context of revitalization works [50] (Figure 1). Site P1 is located in a semi‐confined sector near the upstream portion of the Tibau Channel, characterized by reduced circulation near the Boqueirão Trail; P2 is positioned near Pontal Island, in the central area of the lagoon, which exhibits partial isolation from direct inflow sources; P3 corresponds to the discharge zone of the Cafubá Stream, an area subjected to high pollutant loads associated with mixed land use, irregular urban occupation, and deficient sanitation infrastructure; P4 is situated adjacent to the Camboatá Channel, near Modesto Island, and reflects the influence of domestic effluents from peripheral neighborhoods and proximity to mangrove remnants and P5 lies close to the Tibau Channel mouth, an area under comparatively higher tidal exchanges and increased exposure to urban runoff and recreational activities.

**FIGURE 1:**
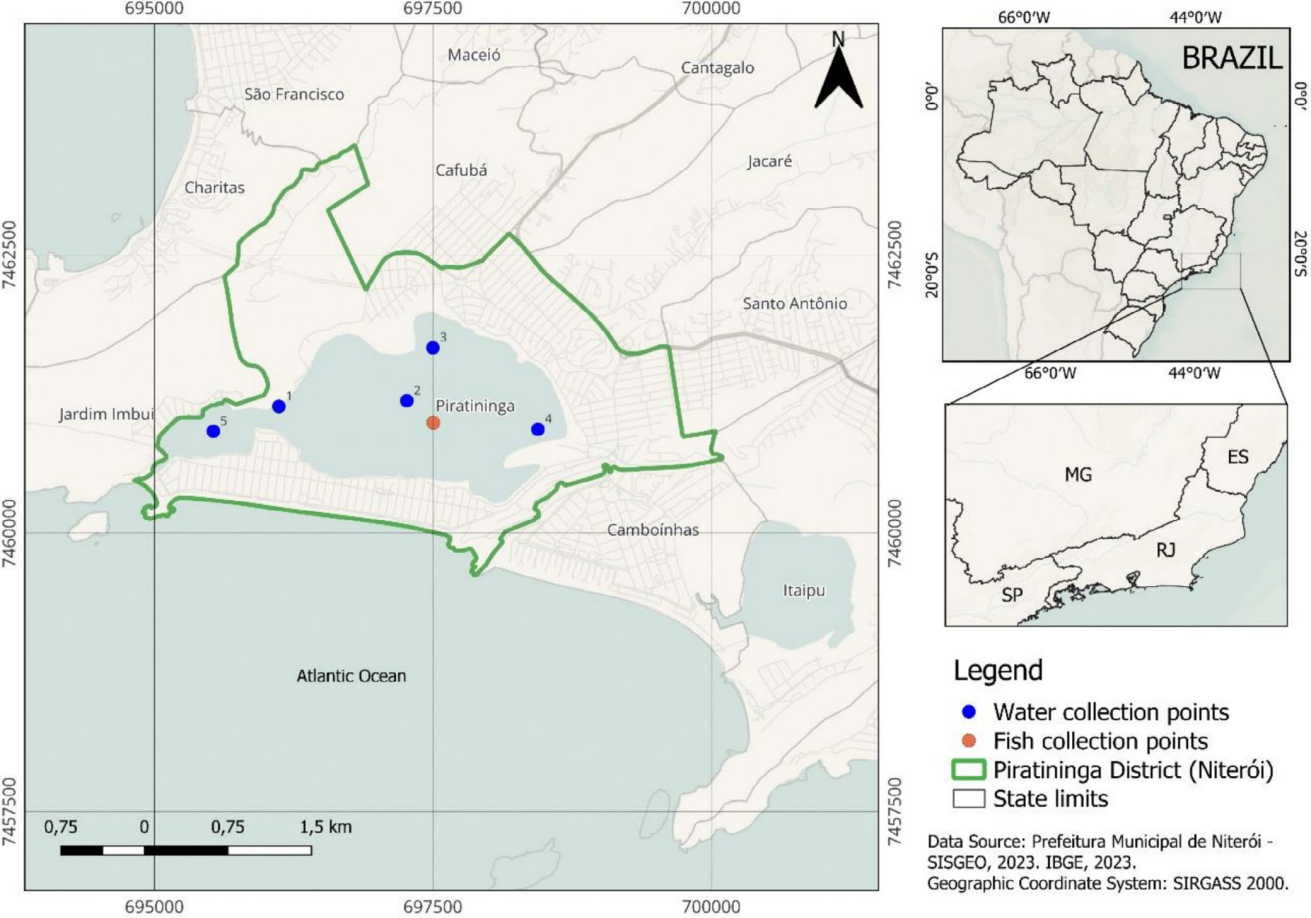
Geographic location of Piratininga Lagoon (PIR), Niterói, Rio de Janeiro,RJ, Brazil. Blue symbols (P1–P5) correspond to water sampling locations, and the orange symbol indicates the fish sampling location. The Piratininga district boundary (green) and regional insets are shown for reference.

### 2.2 Surface Water and Nile Tilapia Sampling

Surface water samplings were carried out at the five predefined stations (P1–P5) (Figure 1) for physicochemical assessments. At each site, approximately 10 L of surface water were collected in high‐density polypropylene containers during four sampling campaigns conducted between 2022 and 2023, encompassing different hydrological periods and pollutant fluxes. Campaigns performed in August 2022 and May 2023 represented the dry season (winter and autumn), and those conducted in November 2022 and February 2023 represented the rainy season (spring and summer). The rainy season is typically associated with increased precipitation, surface runoff, and sediment resuspension, whereas the dry season is marked by reduced water volume and longer water body residence times.

Field measurements were obtained for hydrogen potential (pH), dissolved oxygen (DO), temperature, turbidity, electrical conductivity (EC), salinity, and total dissolved solids (TDS) using a multiparameter probe (Horiba U‐50, Kyoto, Japan). Water samples destined for laboratory analyses were transported under controlled conditions and processed according to APHA guidelines [51]. Thermotolerant coliforms, chemical oxygen demand (COD), ammoniacal nitrogen (method 4500‐NH_3_‐F), and total phosphorus concentrations (Hach TNT 843 and 844 kits) were determined in accordance with the protocols described in Standard Methods for the Examination of Water and Wastewater [51]. For metal and metalloid determinations, subsamples of each water sample (50 mL) were acidified (1 mL) immediately after collection with sub‐boiled double‐distilled nitric acid (65% v/v; Vetec, Rio de Janeiro, Brazil) and stored frozen (−20 ^o^C) until instrumental analysis.

Nile tilapia were collected from a single fixed capture site within Piratininga Lagoon (Figure 1), selected as representative of integrated exposure conditions. Specimens were obtained through 10 casts of a net measuring 12.0 m in perimeter and 30.0 mm in mesh size. The standardized sampling efforts across seasons ensured Nile tilapia size comparability among sampling periods.

Spatial variations in fish metrics were not assessed, as the sampling design focused on temporal (seasonal) and biological (sex‐related) differences. A total of 57 fish were obtained, 30 in the dry season and 27 in the rainy season, comprising 28 females and 29 males. Species identification was conducted according to Eschmeyer et al. [52]. The specimens were placed in 45 L insulated containers filled with local water and maintained under continuous aeration using an air pump. Blood was drawn from the caudal vein using BD Ultra‐Fine syringes (50 U, 12.7 mm × 0.33 mm; New Jersey, USA), pre‐treated with sodium heparin (150 IU mL^−1^), transferred to microtubes, chilled during transport, and centrifuged at the laboratory at 1200 × *g* for 10 min to obtain plasma. The obtained plasma was used for VTG analyses, indirectly estimated through alkaline phosphatase (ALP) activity, as VTG contains alkali‐labile phosphate groups. Following blood collection, fish were euthanized by immersion in ice (0°C to −4°C) for 10 min until no opercular movement, followed by decapitation, in accordance with AVMA Guidelines [53], sexed, measured (total length), weighed (total weight), and dissected. Liver and muscle tissue were stored at −80 ^o^C for further analysis. All animal experiments were conducted with approval from the Chico Mendes Institute for Biodiversity Conservation (SISBIO license no. 87698‐1), the National System for the Management of Genetic Heritage and Associated Traditional Knowledge (SISGEN license no. A5C3171), and the Rio de Janeiro State University Ethics Committee on the Use of Animals (protocol no. 021/2018).

Tilapia liver and gonads were weighed to calculate two organosomatic indices, the Hepatosomatic Index and the Gonadosomatic Index, calculated according to Equations (1) and (2) [54]. Considering that only maturing or mature individuals are associated with reproductive events, the GSI was calculated only for adult individuals.

(1) $$\mathrm{HSI}=\frac{\mathrm{Wh}}{\mathrm{Wt}}\times 100$$

(2) $$\mathrm{GSI}=\frac{\mathrm{Wg}}{\mathrm{Wt}}\times 100$$

where *Wh*/*Wg* comprises the weight of the analyzed organ (liver/gonad, g) and *Wt,* total fish weight (g).

The Fulton condition factor (*K*), a general health state indicator, was also calculated [55] (Equation 3):

(3) $$K=\frac{\mathrm{Wt}}{{\mathrm{Lt}}^{3}}\times 100$$

where *Wt* is the total fish weight (g) and Lt, total length (cm).

### 2.3 Antioxidant System Processing

For antioxidant system assessments, about 50 mg of tilapia liver and muscle, respectively, were homogenized in 0.1 mol L^−1^ sodium phosphate buffer (pH 6.5), containing 0.25 mol L^−1^ sucrose and 1 mmol L^−1^ EDTA, at a 1:4 (m/v) ratio and centrifuged at 20,000 g for 30 min at 4°C (Eppendorf 5430R centrifuge, Hamburg, Germany). The resulting supernatants were used to quantify total proteins according to Lowry [56] modified by Peterson [57] as a normalization factor for some of the analyzed oxidative stress biomarkers, described in the next section.

Liver and muscle extracts were diluted at different ratios depending on the biomarker. Superoxide dismutase, MT and reduced glutathione (GSH) were analyzed using liver extracts diluted 50× and muscle extracts diluted 40× with ultrapure water. Glutathione S‐transferase activity was measured in undiluted samples for both tissues. For total antioxidant capacity (TAC), extracts were diluted 10× for liver and 5× for muscle. Liver extracts were diluted 5× while muscle extracts were diluted 2× for lipid peroxidation (LPO) and protein carbonylation (PTC) assessments.

### 2.4 Antioxidant Biomarker Determinations

Superoxide dismutase activity was evaluated using a commercial kit from Cayman Chemical Company (USA), based on a colorimetric method for total SOD (Cu/Zn, Mn, and Fe SOD isoforms) by detecting superoxide radicals through tetrazolium salt at 450 nm employing an INNO microplate reader (LTek Co., Republic of Korea).

Glutathione S‐transferase activity was determined by monitoring the formation of S‐(2,4‐Dinitrophenyl)‐glutathione (GS‐DNB), an intermediate product formed from the reaction between GSH and 1‐chloro‐2,4‐dinitrobenzene, according to Habig et al. [58] at 340 nm using a UV‐M51 Vis spectrophotometer (Bel Engineering, Italy). Enzymatic activity was expressed as international units (U = μmol L^−1^ min^−1^), normalized by total protein content (U mg^−1^ ptn).

Reduced glutathione levels were quantified according to Wilhelm Filho et al. [59]. Briefly, samples and calibration standards were incubated with 0.25 mmol L^−1^ 5,5′‐dithiobis (2‐nitrobenzoic acid) (DTNB) at a 1:1 ratio for 15 min and absorbances were measured at 412 nm using the INNO microplate reader.

The TAC of the samples was quantified using Trolox as the analytical curve standard, and reduction with an [2,2′‐azinobis (3‐ethylbenzothiazoline‐6‐sulfonic acid) diammonium salt] and potassium persulfate (K_2_S_2_O_8_) solution, according to Pellegrini et al. [60] at 734 nm using an INNO microplate reader. Results were expressed as mmol mg^−1^ ptn.

Metallothionein extraction was carried out using about 150 mg of each sample, according to Erk et al. [61]. The extraction buffer consisted of 0.02 mol L^−1^ Tris (pH 8.6), 5 μL of phenylmethanesulfonyl fluoride, and 0.01% β‐mercaptoethanol. Samples were centrifuged at 20;000 × *g* for 60 min at 4°C on an Eppendorf 5430R centrifuge (Hamburg, Germany). The resulting supernatants underwent thermal extraction at 70°C for 10 min, followed by another centrifugation under the same conditions for 30 min and supernatant separation from the pellets. Quantification followed the Ellman [62] method, using a 1 mol L^−1^ HCl solution containing 0.004 mol L^−1^ EDTA, 2 mol L^−1^ NaCl, and 0.43 mol L^−1^ DTNB, with a 30 min incubation and determinations at 412 nm on the INNO microplate reader. Results were expressed as μmol mg^−1^ ptn, with a stoichiometric conversion factor of 1 mol MT equivalent to 20 mol GSH, as described by Kägi [63]. The treated supernatants were also used for the determination of metals associated to MT, to assess potential MT detoxification routes.

### 2.5 Oxidative Damage Biomarkers

Protein carbonylation levels were determined using the 2,4‐dinitrophenylhydrazine assay, based on the interaction of this reagent with carbonyl functional groups in oxidized proteins according to Mesquita et al. [64]. To this end, the same supernatant obtained for the SOD and GST assays was employed, using 80 μL of liver and muscle samples previously diluted with ultrapure water. The samples were incubated for 10 min with 10 mmol L^−1^ 2,4‐dinitrophenylhydrazine prepared in 0.5 mol L^−1^ phosphoric acid and absorbances were determined at 450 nm using the INNO microplate reader. Results were expressed as μmol mg^−1^ ptn.

Lipid peroxidation levels were assessed by quantifying malondialdehyde (MDA) levels using thiobarbituric acid, according to Esterbauer and Cheeseman [65]. For sample preparation, about 100 mg of each tissue were homogenized in 0.4 mL (1:4, m/v) of an extraction solution containing 0.5 mL of Triton X‐100, 2.5 mL of 1 mol L^−1^ Tris–HCl (pH 7.5), 2.5 mL of 3 mol L^−1^ NaCl, 0.05 g of sodium dodecyl sulfate, and 0.5 g of sodium deoxycholate. The homogenates were subjected to centrifugation at 1600 × *g* for 10 min at 4°C, followed by incubation at 100°C for 60 min and another centrifugation step under the same conditions. The MDA concentrations were determined spectrophotometrically at 535 nm using the INNO microplate reader, and results were reported as μmol mg^−1^ ptn of MDA.

### 2.6 Vitellogenin Determinations

Plasma VTG was indirectly quantified using the alkali‐labile phosphate (ALP) method, as described by Gagné and Blaise [66] and modified by Hallgren et al. [67]. In this approach, VTG induction was inferred from the quantification of alkali‐labile phosphate levels in plasma, based on the presence of phosphate groups associated with the VTG molecule. Briefly, 100 μL plasma samples were precipitated with 54 μL of 35% acetone (v/v) and centrifuged at 5,000 × *g* for 5 min. The pellets were then treated with 100 μL of 1 mol L^−1^ NaOH at 70°C for 90 min, cooled on ice for 10 min and precipitated with 40 μL of 100% TCA, followed by another centrifugation step at 20,000 × *g* for 5 min. The supernatant aliquots (105 μL) were diluted with 435 μL of distilled water and an equal volume of ≥ 99% 1‐butanol. The PO₄^3−^ quantification was performed using a microplate containing 145 μL of each sample, 70 μL of 1% ammonium molybdate in 2 mol L^−1^ H₂SO₄ and 70 μL of 5% ascorbic acid, incubated for 60 min at 40°C. Absorbances were determined at 630 nm and results were expressed as μg PO_4_^3−^ mL^−1^ in plasma. This method offers an indirect estimate of estrogenic responses, which should be considered as indicative rather than a direct quantification of circulating VTG [68, 69].

### 2.7 Metal and Metalloid Determinations

Metals and metalloids in fish tissues and water samples were determined by inductively coupled plasma mass spectrometry (ICP‐MS) employing a NexIon 300X apparatus (PerkinElmer, Norwalk, CT, USA). Total concentrations were quantified only in muscle tissue, as liver samples did not provide sufficient mass for this analysis, whereas MT‐enriched subcellular metal and metalloid concentrations were determined in both muscle and liver.

For water, the preserved samples containing 1.0 mL of sub‐boiled double‐distilled nitric acid were analyzed without further processing. Concerning fish, total metal and metalloid determinations were carried out using 0.100 g of each tissue, while an equivalent amount of the MT‐enriched subcellular fraction was analyzed for metal and metalloid concentrations, as this fraction represents a major component of the intracellular labile metal pool. Metal and metalloid concentrations in water were expressed as mg L^−1^ and in fish, as mg kg^−1^ wet weight (w.w.).

Fish samples were transferred to sterile polypropylene Falcon‐type 15 mL tubes and subjected to acid digestion overnight with 1.0 mL of subboiled double‐distilled nitric acid (Vetec, Rio de Janeiro, Brazil). Following digestion, each sample was heated on a heating block at 100°C for approximately 6 h [70]. To prevent pressure buildup, the polypropylene screw‐capped Falcon tubes were briefly opened and closed every hour to avoid tube rupture or explosion during sample processing. Following cooling to room temperature, the digested samples were diluted to a final volume of 10 mL with ultra‐pure water.

Metals and metalloids were determined employing external multielement calibration curves, with correlation coefficients always above 0.995. Sample introduction was performed using a Meinhard nebulizer coupled to a cyclonic spray chamber, ensuring stable aerosol generation and signal precision. Instrumental drift and signal fluctuations were monitored and corrected using Rh as an internal standard injected online throughout all measurements at 20 mg L^−1^. Throughout the analytical sequence, Rh signal variations remained within acceptable limits, indicating minimal instrumental drift, and stability was verified through repeated QC samples analyzed at regular intervals. Quality control procedures included strict blank controls, the analysis of three analytical replicates, and repeated measurements of a certified reference material (DORM‐5, fish tissue, NRC, Canada), processed identically to the samples and analyzed in triplicate. Limits of Detection (LOD) and Limits of Quantification (LOQ) were calculated according to the Brazilian National Institute of Metrology, Quality and Technology [71]. The respective LOD and LOQ values for water and fish samples (total and MT‐enriched subcellular fraction) are presented in Table S1. Recoveries ranged from 70% to 114% of the certified values, considered acceptable for the analyzed matrices [72] (Table S2).

### 2.8 Human Health Risk Assessment

To evaluate potential risks associated with tilapia consumption, several human health risk assessment indices were calculated. Calculations were performed separately for boys/men and girls/women across different age groups, as follows: infants (7–12 months old, as fish consumption is recommended from 6 months according to WHO guidelines); children (3–11 years old); and adults (19–75 years old), under consumption scenarios from one to five times a week.

The exposure scenarios (1–5 fish meals per week) were defined to represent a range of consumption patterns, encompassing moderate to relatively high intake frequencies. Nevertheless, consumption rates may vary among specific demographic groups (*e.g*., children, subsistence fishers, or high‐frequency consumers).

Limits and health‐based guidance values regarding metals in foodstuffs and EDI/EWI established by the US Food and Drug Administration (FDA), US Environmental Protection Agency (EPA), the Brazilian ANVISA and the WHO were used for comparison purposes, based on seafood consumption data when available, and in foodstuffs in general when not, considering fish muscle as the edible tissue [73, 74, 75, 76, 77, 78, 79, 80, 81, 82].

Specifically regarding As, three exposure scenarios were considered, concerning inorganic As, which is the toxic form of this metal and has been noted as present ranging from about 1% to 10% of total As in fish muscle: (1) an average scenario, employing the mean concentrations of each element, along with their minimum and maximum values; (2) a best‐case scenario, assuming that only 1% of arsenic (As) in tilapia muscle is in its inorganic (toxic) form; and (3) a worst‐case scenario, where 10% of As is in the inorganic form.

The Estimated Daily Intake (EDI) for each element was determined as follows:

(4) $$\mathrm{EDI}=\frac{C\times \mathrm{FIR}}{\mathrm{BWa}}$$

where *C* is the concentration of the element in tilapia muscle (mg kg^−1^), *F (fish ingestion rate)* comprises the average daily fish intake (0.015 kg day^−1^ for infants of both sexes, 0.025 kg day^−1^ for children of both sexes, and for adults, sex‐specific rates of 0.050 kg day^−1^ for males and 0.045 kg day^−1^ for females) [83] and *BWa* is the reference body weight for each age group (kg g^−1^). To estimate weekly intakes (EWI), the EDI was multiplied by 7. Both EDI and EWIs were compared to age‐specific reference intake limits according to the EFSA [84]; FAO/WHO [85] and USEPA [86], as follows: For infants, tolerable intakes were set as Cd < 0.00083 mg day^−1^ bw, Cu < 1 mg day^−1^ bw, MeHg < 0.0016 mg day^−1^ bw, Se < 0.06 mg day^−1^ bw, and Zn < 7 mg day^−1^ bw. For children, limits were set as Cd < 0.00083 mg day^−1^ bw, Cu < 3 mg day^−1^ bw, MeHg < 0.0016 mg day^−1^ bw, Se < 0.13 mg day^−1^ bw, and Zn < 13 mg day^−1^ bw. For adults, they were Cd < 0.0008 mg day^−1^ bw, Cu < 5 mg day^−1^ bw, MeHg < 0.0016 mg day^−1^ bw, Se < 0.30 mg day^−1^ bw, and Zn < 25 mg day^−1^ bw.

The Target Carcinogenic Risk (TCR) was used to evaluate the lifetime cancer risk due to As and Pb exposure and calculated as:

(5) $$\mathrm{TCR}=\left(\frac{\mathrm{EFR}\times \mathrm{ED}\times \mathrm{FIR}\times C\times \text{CPSO}}{\mathrm{BWa}\times \mathrm{ATc}}\right)\times 10^{-3}$$

where *EFR* is the exposure frequency (days per year); *ED* comprises the exposure duration (2 years for infants, 11 years for children, and 75 years for adults); *FIR* is the daily fish ingestion rate (mg per day); *C* is the As concentration in tilapia muscle (mg kg^−1^); *CPSO* consists of the oral cancer slope factor for inorganic As (1.5 mg kg^−1^ day^−1^), as defined by USEPA [86]; and Pb = 0.0085 mg kg^−1^ day^−1^, as defined by OEHHA [87]; *BWa* is the reference body weight; *ATc* represents an average exposure duration (365 days × year^−1^ per category, minus 6 months, since solid food introduction begins at 6 months of age in infants); and 10^−3^ comprises a unit conversion factor.

To assess non‐carcinogenic risks, the target hazard quotient (THQ) was determined as:

(6) $$\mathrm{THQ}=\left(\frac{\mathrm{EFR}\times \mathrm{ED}\times \mathrm{FIR}\times C}{\mathrm{RfD}\times \mathrm{BWa}\times \mathrm{ATn}}\right)\times 10^{-3}$$

where *RfD* is the reference oral dose (mg g^−1^ per day^−1^) sourced from the EPA's IRIS database, from the OEHHA [87], USEPA [86] and Taylor et al. [88] and *ATn* consists of the average exposure time (365 days × year^−1^ per category, minus 6 months, since solid food introduction begins at 6 months of age for infants). Reference oral doses were 5 × 10^−5^ μg g^−1^ day^−1^ for Ag, 3 × 10^−4^ for inorganic As, 1 × 10^−3^ for Cd, 3 × 10^−3^ for Cr, 5 × 10^−2^ for Cu, 1 × 10^−4^ for Hg, 0.14 for Mn, 3.6 × 10^−3^ for Ni, 5 × 10^−3^ for Se, 9 × 10^−3^ for V, and 0.3 μg g^−1^ day^−1^ for Zn.

The Hazard Index (HI) was determined as the sum of all THQs for elements with an established RfD.

### 2.9 Statistical Analyses

Outliers were removed by the ROUT method, adopting a discovery rate of 1% (*Q* = 1%). Seasonal differences (dry and rainy) in biochemical responses, elemental concentrations, and morphometric parameters were evaluated separately for liver and muscle tissues. Data distribution was examined using the Shapiro–Wilk test. Seasonal comparisons were performed using the Mann–Whitney test. Biomarker data were summarized as medians accompanied by minimum and maximum values, whereas physicochemical water parameters and elemental concentrations were reported as mean values with corresponding standard deviations. The same statistical approach was adopted for total fish length, body mass, sex distribution, and organosomatic indices between seasons.

Associations between metal and metalloid concentrations in fish muscle were examined using Spearman's rank correlation coefficient, considering each seasonal period independently. Correlation matrices were computed using the *rcorr* function from the Hmisc package. Correlation strengths were interpreted according to Bryman and Cramer [89] as very weak (*r* < 0.20), weak (0.20–0.39), moderate (0.40–0.69), strong (0.70–0.89) or very strong (≥ 0.90). Visualization of correlation patterns was carried out using the *corrplot* package, displaying the upper triangular matrices.

The multivariate structure within the dataset was further explored through a principal component analysis (PCA), conducted independently for liver and muscle tissues, in order to identify integrated patterns among oxidative stress biomarkers and metals associated with the MT‐enriched subcellular fraction for different seasonal conditions. Data suitability was confirmed using Bartlett's test of sphericity (*p* ≤ 0.05) and the Kaiser–Meyer–Olkin (KMO) measure of sampling adequacy, with KMO values below 0.50 excluded from the analysis. The PCA was performed using the *prcomp* function, and interpretation was based on eigenvalues, explained variance, variable loadings and squared cosine (cos^2^) values. All univariate statistical analyses were carried out using GraphPad Prism (version 8), while multivariate procedures were conducted in RStudio (version 2025.05.0 + 496). Statistical significance was established at *p* ≤ 0.05.

## 3 Results and Discussion

### 3.1 Water Quality Parameters

Water surface samples were analyzed for 15 physicochemical and biological indicators (Table S3). Mean water temperature and salinity values were 25.9°C ± 4.4°C and 16.2 ± 4.2 PSU, respectively. The pH values ranged from 8.2 to 9.9 (mean 9.1 ± 0.5), with the lowest (8.2) and the highest (9.9) values observed at P1 during the dry season. Dissolved oxygen levels varied between 5.1 and 12.7 mg L^−1^ (mean 8.8 ± 2.3 mg L^−1^), while oxygen saturation (DO a%) ranged from 75.7% to 165% (mean 118.3 ± 26%), with the lowest readings frequently observed at sampling sites P3 and P4 during the rainy season. Reduced values suggest possible organic enrichment and may compromise aquatic life under low DO, high turbidity, or elevated TDS conditions, commonly associated with eutrophication and oxygen depletion caused by the high oxygen demand required for the breakdown of organic matter in effluents [90]. All measurements remained above the minimum threshold values established by Brazilian environmental legislation (CONAMA) [91]. Turbidity varied from 10.7 to 65.8 NTU (mean 31.1 ± 15.2 NTU), with the maximum value recorded at P3 and the minimum at P4 during one rainy season. Total dissolved solids ranged from undetected to 174 g L^−1^, exceeding the limits of Brazilian CONAMA 357/2005 guidelines of 0.5 g L^−1^ in all campaigns, except the first dry campaign.

Average phosphorus (P) and total nitrogen (TN) values were 0.8 ± 0.3 mg L^−1^ and 5.8 ± 7.9 mg L^−1^, respectively. Domestic wastewater represents a major source of inorganic nutrients, such as nitrogen (NH_3_‐N) and phosphorus (PO_4_‐P), significantly contributing to the nutrient loads of receiving aquatic environments [92]. The high nutrient levels observed in this study align with Acioly et al. [93], who reported high nitrogen and chlorophyll concentrations in urban stretches of the Tocantins River, particularly at sites influenced by domestic sewage. During the dry season, BOD and COD levels were 21.6 ± 3.2 mg L^−1^ and 107.9 ± 16.2 mg L^−1^, respectively, lower than in the rainy season (BOD: 38.04 ± 48.9 mg L^−1^, COD: 190.2 ± 244.7 mg L^−1^). The data were compared with Brazilian standards for surface water intended for recreational, fishing, and bathing, with a BOD limit of < 5 mg L^−1^ for Class 2 waters [91]. In both seasons, BOD values were significantly above this threshold. Although COD is not explicitly regulated by CONAMA Resolution 357/2005 for Class 2 waters, it can serve as a complementary indicator in environmental assessments, particularly in areas affected by organic pollution [94].

Concerning the microbiological analyses, thermotolerant coliforms (CtT) ranged from 2419.6 to 3 410 000 MPN 100 mL^−1^, with the highest value observed during the first dry sampling campaign at P1. *Escherichia coli* concentrations ranged from non‐detected samples to 6140 MPN 100 mL^−1^, with the maximum value also recorded during the second dry campaign at P3. It indicates significant seasonal variations in surface water quality (*p* ≤ 0.05). Thermotolerant coliforms, produced in the intestines of warm‐blooded animals, are key fecal contamination indicators due to their persistence in aquatic environments [94, 95], and both *Escherichia coli* and thermotolerant coliforms are widely used as cost‐effective sewage pollution indicators [96]. According to the Brazilian CONAMA Resolution 357/2005, Class 2 surface waters must not exceed 1000 MPN 100 mL^−1^ of total coliforms and 200 MPN 100 mL^−1^ of thermotolerant coliforms. The resolution considers thermotolerant coliforms as proxies for fecal coliforms, while *E. coli* , though not mentioned, is commonly used in international standards.

### 3.2 Surface Water Metal and Metalloid Levels

The metal and metalloid results in surface water are presented in Table S4, expressed as seasonal mean values across all sampling points. A greater variety of metals and metalloids was detected during the dry season, being 12 elements identified, while seven were determined in the rainy season. Arsenic, Mg, Mn, Mo, Rb, Ti, and V were present in all samples during both seasons. During the dry season, Fe concentrations ranged from 1.06 to 1.45 mg L^−1^ (mean 1.20 ± 0.13 mg L^−1^). Mercury was detected at low levels, ranging from 0.005 to 0.007 mg L^−1^ (mean 0.006 ± 0.001 mg L^−1^). Nickel was present at three sampling points (P1, P2, and P3). Lead and Zn were also detected during the dry season, with mean concentrations of 0.002 ± 0.002 mg L^−1^ and 0.121 ± 0.005 mg L^−1^, respectively.

These findings suggest that the dry period leads to heightened metal and metalloid water column mobilization and availability, probably due to reduced water volume, which limits contaminant dilution and favors the concentration of elements previously diluted or adsorbed to particles [97, 98, 99]. In tropical coastal regions, such as South America, the dry season is typically characterized not only by low rainfall but also by high temperatures and increased evaporation [100], which intensify solute retention and concentration in aquatic environments [101]. Furthermore, continuous physical sediment disturbance may occur due to anthropogenic activities, such as navigation and fishing, or natural forces like wind action, in lentic and semi‐lentic shallow systems [102]. These processes can facilitate the remobilization of sediment‐bound metals into the water column, reinforcing the patterns observed herein and supporting another assessment [103]. Additionally, the higher detection of metals and metalloids at sampling points P1, P2, and P3 can be explained by their environmental context, as P1 and P2 are located in semi‐confined or semi‐isolated sectors, which favor contaminant accumulation, while P3 receives direct inputs of untreated urban effluents.

### 3.3 Morphometric Tilapia Indices

Nile tilapia total length and body weight are summarized in Table S5. While total weight did not vary significantly by season, total length differed significantly between campaigns (*p* ≤ 0.05), with smaller individuals recorded during the dry period (winter). Some seasonal differences in organosomatic indices were also observed (Table 1). During the rainy season, no significant differences were found between males and females for GSI, HSI, or *K*, while a significant sex‐specific difference was detected in the dry season for GSI (*p* ≤ 0.05), which may reflect not only reproductive activity but also the potential influence of environmental contamination [104, 105].

**TABLE 1:** Organosomatic indices (means ± standard deviations) calculated for Nile Tilapia ( *Oreochromis niloticus* ) during the dry (winter and autumn) and rainy season (spring and summer) of 2021 at Piratininga Lagoon, Rio de Janeiro, southeastern Brazil.

	Dry season					Rainy season				
	Female		Male		Mean ± SD	Female		Male		Mean ± SD
	Median (min–max)	Mean ± SD	Median (min–max)	Mean ± SD		Median (min–max)	Mean ± SD	Median (min–max)	Mean ± SD	
GSI	**1.12 (0.26–3.33)**	**1.23 ± 0.94**	**0.24 (0.03–0.59)**	**0.24 ± 0.17**	**0.73 ± 0.80**	0.80 (0.24–3.49)	1.12 ± 0.895	0.72 (0.15–1.27)	0.727 ± 0.297	0.93 ± 0.69
HSI	0.13 (0.06–0.35)	0.15 ± 0.07	0.17 (0.04–0.50)	0.17 ± 0.11	0.16 ± 0.10	0.09 (0.04–0.49)	0.154 ± 0.140	0.17 (0.09–0.57)	0.192 ± 0.124	0.17 ± 0.13
K	2.08 (1.70–2.51)	2.11 ± 0.25	1.94 (1.70 2.46)	1.97 ± 0.19	11.30 ± 5.6	1.97 (1.6–2.67)	2.03 ± 0.268	1.89 (1.30–2.41)	1.92 ± 0.282	8.40 ± 2.61

*Note:* Values in bold indicate statistically significant differences according to the Mann–Whitney test (*p* < 0.05).

The determined somatic and reproductive indices offer integrated insights into energy allocation, reproductive investment, and endocrine function throughout organismal reproductive cycles [35]. The GSI reflects gonadal development and is widely used to assess reproductive readiness and spawning periods [54], with peaks typically occuring during late vitellogenesis and final oocyte maturation, especially in females [106]. The HSI, associated with hepatic metabolic activity and energy storage, often increases in parallel with VTG synthesis, which is regulated circulating estradiol‐17β (E2) [107, 108]. As oocytes grow, both GSI and HSI increase along with E2 plasma levels [109]. The *K*, which reflects somatic energy reserves, tends to be higher in well‐nourished fish with lower metabolic stress, while lower values may result from energy depletion due to poor food availability or the energetic cost of detoxification under contaminant exposure [106, 109, 110].

The effects of pollutants, including several metals and metalloids and other endocrine disruptors, on fish reproductive indices are well documented, with details reviewed by Chakraborty [105], indicating that Hg, Pb, Cr, and Cd may act as EDC in fishes, affecting brain neurotransmission, gonadal structure/function, and modulating reproduction‐related gene expression, similar to other studies [35, 36, 107, 111]. Such compounds may interfere with steroid hormone synthesis, impairing gametogenesis through mechanisms like aromatase inhibition, gonadal cell damage, and apoptosis [112]. Furthermore, EDC can also impair gonadal development, delay puberty, alter germ cell populations, and induce intersex conditions [106, 113]. Disturbances in endocrine functions are frequently reflected in altered GSI values. Herein, females exhibited a significant increase in GSI during the dry season, whereas males showed a marked reduction. As female GSI values naturally vary with reproductive stage, this pattern may reflect physiological seasonality. Higher metal concentrations were observed in water and tissues during the dry campaign (Tables S4 and S6), consistent with contaminant exposure, although no causal link was noted. This contrasting pattern may indicate sex‐specific physiological responses to environmental contaminants and can be indicative of endocrine impairment associated with metal and metalloid exposure [106]. Several studies support this interpretation, demonstrating strong correlations between hormonal regulation [106], gonadal development [35, 113], fish maturation [112], and changes in organosomatic indices in contaminated environments [107, 114, 115].

### 3.4 Vitellogenin Induction

Vitellogenin responses were inferred from plasma alkali‐labile phosphate (PO₄^3−^) levels in male fish (Figure 2). Statistically significant ALP differences were observed when comparing males and females within each season (*p* ≤ 0.05). During the dry season, females showed a median of 5.74 μg PO₄^3−^ mL^−1^ plasma (range: 0.62–71.16), while males presented 10.61 μg PO₄^3−^ mL^−1^ (range: 2.00–53.19). In the rainy season, females had a median of 16.42 μg PO₄^3−^ mL^−1^ plasma (range: 3.76–54.89), and males 7.98 μg μg PO₄^3−^ mL^−1^ (range: 4.51–36.54). Conversely, the male–female VTG ratio was 1.85 during the dry season and 0.49 in the rainy season, indicating an atypical patternin which males exhibited higher relative VTG concentrations than females during the dry period. The observed VTG pattern agreed with the GSI findings, which also indicated a significant sex effect in the same season.

**FIGURE 2:**
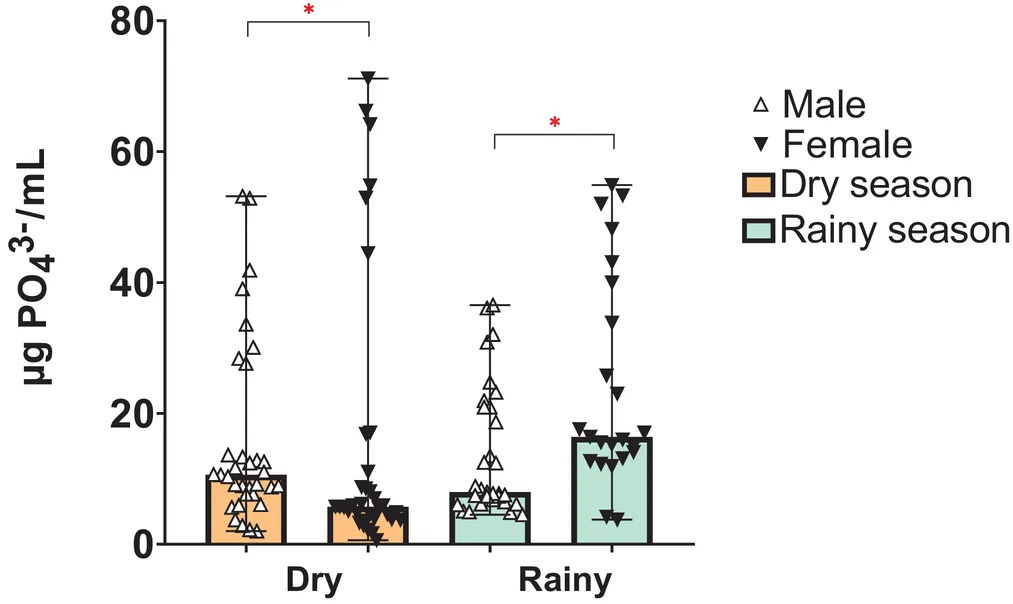
Plasma alkali‐labile phosphate (ALP) concentrations in Nile tilapia ( *Oreochromis niloticus* ) as an indirect vitellogenin (VTG), grouped by sex (∆ male; ▼ female) and season (dry and rainy). Data are presented as medians, with whiskers indicating the maximum and minimum values. Asterisks (*) denote significant differences in the comparison between sexes within the same season (Mann–Whitney test, *p* < 0.05).

Vitellogenin is a key lipophosphoprotein involved in vitellogenesis, normally synthesized in the liver of females in response to circulating 17β‐estradiol [108]. Once produced, it is transported through the bloodstream to the ovaries and incorporated into developing oocytes [116]. However, this process can be impaired by internal factors, such as hormonal imbalances or external factors, including environmental pollutants [37]. Previous studies have shown that fish with reproductive failure exhibit reduced circulating levels of 17β‐estradiol [109] and lower VTG mRNA expression in liver and ovarian tissues [38].

The detection of VTG in males may indicate exposure to environmental endocrine‐disrupting compounds with estrogenic activity [37]. Previous studies have reported VTG induction in males exposed to metal‐contaminated environments [35, 36, 117]. The inverted male–female VTG ratio in the dry season observed herein co‐occurred with higher absolute metal and metalloid levels and high antioxidant/oxidative‐stress endpoints (SOD, GST, MT, LPO, PTC), particularly in males. This pattern is consistent with endocrine modulation under environmental stress, but should not be interpreted as evidence of direct causality. Seasonal hydrological conditions, such as reduced dilution, longer residence time, salinity and temperature shifts, and contaminant resuspension, may enhance the bioavailability of metals, metalloids, and other stressors, potentially influencing endocrine‐related pathways, including steroidogenesis [118].

Importantly, although this period did not coincide with the highest detectable metal and metalloid concentrations in surface water, the presence of other unmonitored contaminants (such as pharmaceuticals, personal care products, and pesticides) cannot be ruled out, especially considering that recent studies identified the occurrence of EDCs in the same study area [46, 119, 120, 121]. Additionally, female VTG often declines outside reproductive peaks [122] and tilapia reproduction, though year‐round in Brazil, is more frequent during milder‐temperature periods [123], which may partly explain lower female VTG levels in the dry season and, consequently, a high male‐to‐female VTG ratio.

Herein, VTG responses were inferred using the alkali‐labile phosphate method, which provides an indirect estimate of estrogenic responses rather than a direct quantification of circulating VTG. Consequently, the observed patterns should be interpreted as indicative of endocrine‐related alterations, rather than conclusive evidence of endocrine disruption. Together, these findings suggest that the male–female VTG patterns observed reflect the combined influence of seasonal environmental conditions, chemical mixtures, and biological variability typical of wild fish under field conditions.

### 3.5 Nile Tilapia Metal and Metalloid Accumulation

Total metal and metalloid concentrations in Nile tilapia are represented in Table S7. Total concentrations were quantified only in muscle tissue, as liver samples did not provide sufficient mass for this analysis, whereas subcellular metal and metalloid concentrations were determined in both muscle and liver. Total concentrations in muscle are illustrated in Figure 3, expressed as mg kg^−1^ wet weight (w.w.), which did not differ significantly between dry and rainy seasons (*p* > 0.05). Both seasons showed the same suite of detected elements (As, Co, Cu, Fe, Mn, Ni, Pb, Rb, Ti, V, and Zn), except Mo, which was detected only in the dry season. Cadmium was below the Limit of Detection (LOD) in all surface water samples and in the total muscle fraction, but was detected at low frequency in the MT‐enriched subcellular fraction. Metal and metalloid concentrations in the MT‐enriched subcellular fraction, expressed in mg kg^−1^ w.w., are detailed in Table 2, for both tissues. Figure 4 presents the relative amounts (%) of total metals and metalloids minus the MT‐enriched fraction (depicted in black) and the MT‐enriched subcellular metal and metalloid fraction (depicted in white) in muscle tissue, in which both fractions when summed total 100%. The LOD and LOQ for each element are listed in Table S1.

**FIGURE 3:**
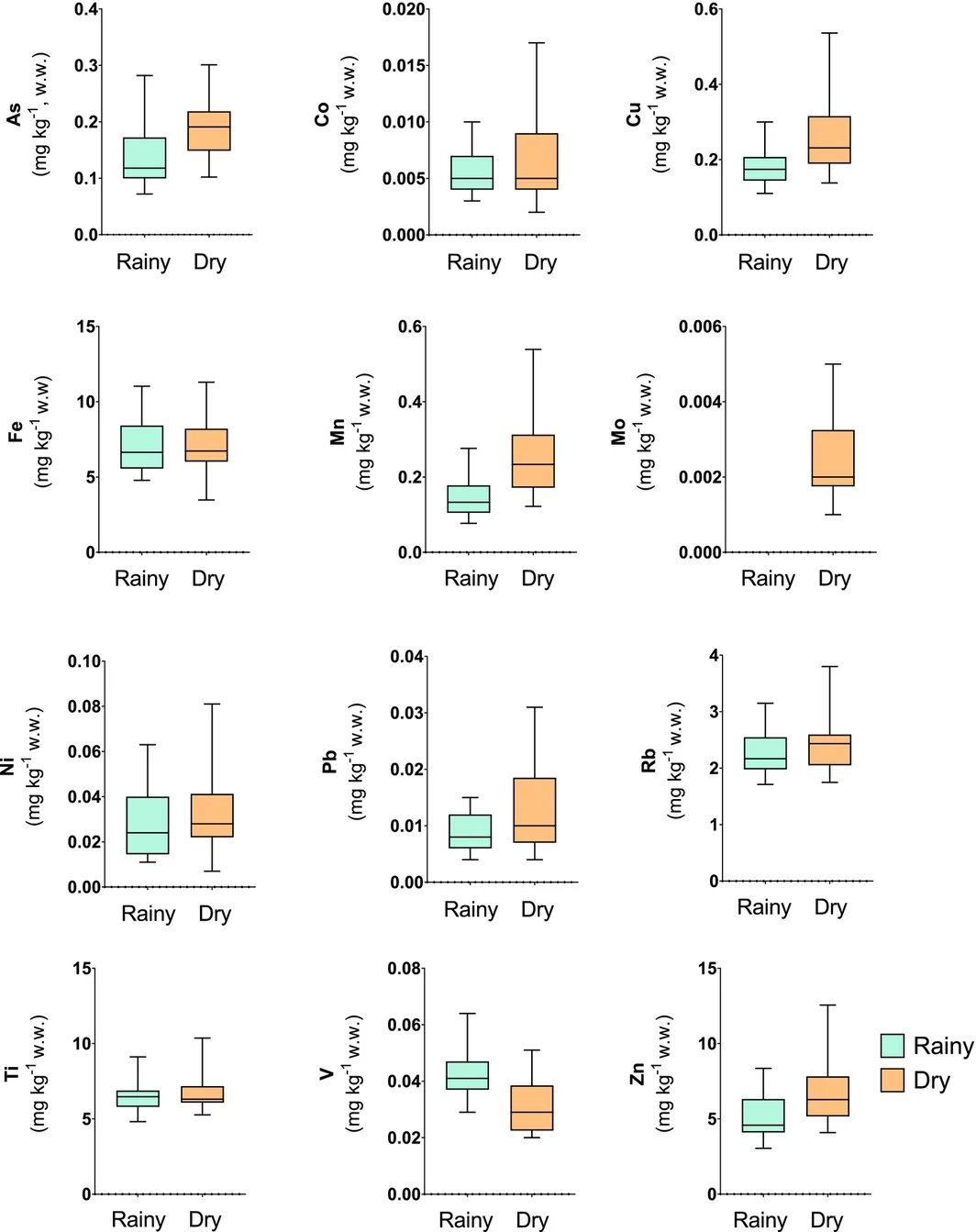
Total metal and metalloid concentrations (mg kg^−1^ wet weight) in the muscle tissue of Nile tilapia ( *Oreochromis niloticus* ) from Piratininga Lagoon, Rio de Janeiro, southeastern Brazil. Data sets in light blue indicate rainy period (spring/summer), while data sets in light orange indicate dry period (autumn/winter). Horizontal lines within the boxplots show medians; the top line indicates the 75^th^ quartile and the bottom line, the 25^th^ quartile. The bounds indicate the maximum and minimum ranges, with the vertical lines extending from the 10^th^ to the 90^th^ percentile.

**TABLE 2:** Intracellular concentrations of metals and metalloids (mg kg^−1^) in Nile tilapia ( *Oreochromis niloticus* ) liver from Piratininga Lagoon, Rio de Janeiro, southeastern Brazil.

	Element	Dry season, mg kg^−1^ (w.w.)	Rainy season, mg kg^−1^ (w.w.)
		Median (min–max)	Median (min–max)
Liver	As	**0.461 (0.406–0.461)**	**0.261 (0.081–0.534)**
	Cd	0.006	< LOQ
	Co	**0.115 (0.060–0.647)**	**0.060 (0.020–0.538)**
	Cu	0.77 (0.170–146.0)	0.31 (0.04–1.64)
	Fe	133 (8.05–194)	66 (32.5–230)
	Hg	0.018 (0.016–0.020)	< LOQ
	Mn	0.196 (0.094–0.338)	0.190 (0.064–1.18)
	Mo	0.031 (0.022–0.051)	< LOQ
	Ni	0.108 (0.070–0.270)	0.132 (0.05–0.34)
	Pb	0.033 (0.026–0.045)	< LOQ
	Rb	**3.46 (3.40–4.92)**	**0.55 (0.01–1.90)**
	Se	**0.354 (0.177–0.506)**	**29.3 (7.345–42.15)**
	Ti	5.31 (3.85–7.88)	4.29 (0.21–9.54)
	V	0.20 (0.168–0.297)	0.273 (0.124–0.485)
	Zn	5.52 (1.55–8.66)	6.38 (0.93–18.39)
Muscle	As	**0.503 (0.281–0.561)**	**0.351 (0.084–0.648)**
	Cd	< LOQ	0.005 (0.003–0.013)
	Co	**0.055 (0.022–0.164)**	**0.025 (0.002–0.250)**
	Cu	0.118 (0.067–0.131)	0.166 (0.051–0.622)
	Fe	**4.92 (4.22–6.41)**	**8.24 (4.27–39.63)**
	Hg	0.039 (0.018–0.102)	0.014 (0.014–0.030)
	Mn	0.204 (0.104–0.30)	0.142 (0.022–0.620)
	Mo	0.020 (0.009–0.050)	< LOQ
	Ni	0.091 (0.055–0.178)	0.116 (0.033–4.48)
	Pb	0.024 (0.021–0.045)	< LOQ
	Rb	**3.64 (2.49–6.30)**	**0.268 (0.017–1.32)**
	Se	**0.46 (0.010–1.06)**	**15.6 (3.37–23)**
	Ti	5.58 (4.52–7.59)	5.78 (0.332–6.59)
	V	0.131 (0.103–0.160)	0.126 (0.082–0.202)
	Zn	2.30 (1.04–9.28)	5.31 (0.440–30)

*Note:* Values are expressed as median (minimum and maximum) values. < LOQ indicates samples below the limit of quantification. Values in bold indicate statistically significant differences according to the Mann–Whitney test (*p* < 0.05).

**FIGURE 4:**
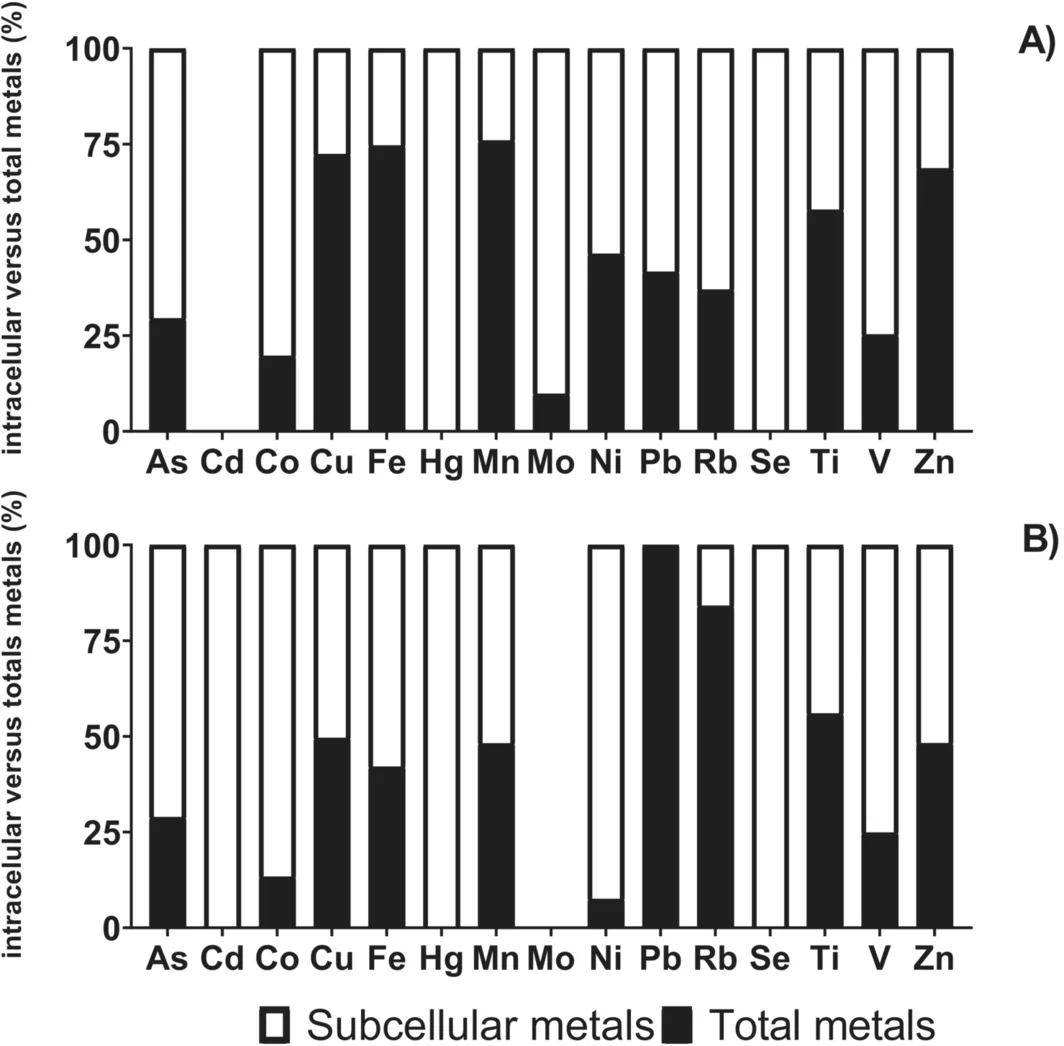
Relative proportions (%) of total metal and metalloid concentrations minus the MT‐enriched fraction (black) and subcellular (white) metal and metalloid concentrations in the muscle tissue of Nile tilapia ( *Oreochromis niloticus* ) from Piratininga Lagoon (PIR) during the dry (A) and rainy (B) seasons. Bars represent the percentages (%) of each fraction grouped by season, which summed total 100%. Bars not shown indicate elements that were not detected.

Concerning total metal and metalloid concentrations (Figure 2), during the rainy period, all elements were present in 100% of specimens. In the dry season, detection frequencies were 48.3% for Mo (14/29) and 93.1% for Pb (27/29); all other elements were found in all samples. Although no statistically significant seasonal differences were observed, Fe medians were slightlyhigher in the rainy than in the dry season (6.72 vs. 6.64 mg kg^−1^). Manganese, Ni, Pb, Rb, and Zn were higher in the dry season (medians: Mn = 0.234, Ni = 0.028, Pb = 0.010, Rb = 2.43, Zn = 6.28 mg kg^−1^ w.w.) than in the rainy season. In contrast, Ti and V were higher in the rainy season compared to in the dry season (medians: Ti = 6.47 [4.81–9.12] w.w., V = 0.041 mg kg^−1^ w.w. [0.029–0.064]).

Even though metal and metalloid concentrations remained within the safety limits established by WHO/FAO for fish consumption [124], the detection of several toxic elements indicates potential human health risks. In this sense, the occurrence of Ni, Pb, and Ti may reflect inputs from urban activities, such as construction debris, industrial waste, and domestic or industrial effluents, which are known to release these metals into aquatic environments [125, 126].

In contrast, concentrations in the MT‐enriched subcellular fraction showed statistically significant seasonal variations (*p* ≤ 0.05), particularly in hepatic tissue, for As, Co, Rb, and Se. Arsenic levels were 76.6% higher during the dry season (median: 0.461 mg kg^−1^ w.w. range: 0.406–0.461 mg kg^−1^ w.w.), with similar patterns noted for Co and Rb, which increased by 91% and 532.5%, respectively. Selenium, however, showed an opposite trend, reaching the highest concentration in the rainy season (29.3 mg kg^−1^ w.w., 7.34–42.1). Additionally, some elements such as Mo (0.031 mg kg^−1^ w.w., 0.022–0.051), Cd (0.006 mg kg^−1^ w.w., single sample), Hg (0.018 mg kg^−1^ w.w., 0.016–0.020) and Pb (0.033 mg kg^−1^ w.w., 0.026–0.045) were only detected in the liver during the dry season, suggesting episodic or seasonal exposure events, similar to the findings reported by El‐Agri et al. [127] on increased biomarker responses during winter, and Maraschi et al. [26], who reported prolonged metal retention following short‐term exposures. In muscle, statistically significant seasonal variations were observed for As, Co, Fe, Rb, and Se (*p* ≤ 0.05). While Cd was detected during the rainy season, Mo and Pb appeared only in the dry season. Arsenic concentrations increased by 43.3% and Co by 122.4% during the dry season, and Rb reached its highest median value in this period (3.64 mg kg^−1^, 2.49–6.30). On the other hand, Fe was 67.5% higher in the rainy season (median: 8.24 mg kg^−1^, range: 4.27–39.63) and Se again reached its highest concentration during this period (15.6 mg kg^−1^, 3.37–23). While the MT‐enriched subcellular metal and metalloid fraction constitutes a major reservoir of labile elements, it does not represent the full spectrum of intracellular metal/metalloid pools, since elements bound to non‐MT cytosolic ligands and to organelles also contribute to cellular regulation and toxicity pathways, but were not quantified herein [23, 26].

The relative distribution of both metal and metalloid fractions in tilapia muscle (Figure 4) revealed distinct patterns among elements and seasons. Arsenic, Co, Hg, and V w predominantly associated with the MT‐enriched subcellular fraction across both seasons, suggesting efficient cellular sequestration mechanisms, likely through complexation with insoluble fractions or binding to organelles, reducing their immediate bioavailability and toxicity [26]. Although subcellular sequestration reduces immediate/acute metal and metalloid toxicity, it may not eliminate long‐term risk [26]. Under physiological or metabolic stress, remobilization of stored elements may occur, potentially increasing intracellular bioavailability and leading to chronic effects over time [128].

In contrast, Mn, Ti, and Zn concentrations were higher in the total fraction, particularly during the dry season, indicating limited intracellular compartmentalization and possibly recent exposure or accumulation [10]. These elements may pose greater ecotoxicological risks due to their increased mobility and potential interaction with cellular targets [10]. Seasonal differences were also evident, with Cd and Mo presenting higher MT‐enriched subcellular fraction in the rainy and dry period, respectively, which may reflect reduced dilution and enhanced retention under lower hydrodynamic conditions [129]. These findings support the hypothesis that metal and metalloid behavior in fish is influenced not only by environmental availability but also by organismal capacity for detoxification and storage, which can vary across seasons and influence biomarker responses [130].

### 3.6 Human Health Risk Assessment

The presence of metals and metalloids in edible tissues, such as muscle, raises potential human health concerns, especially among populations that consume fish regularly as a protein source [45]. Data concerning metal and metalloid risks were first analyzed separately for the rainy and dry seasons and then grouped.

#### 3.6.1 Comparison to Maximum Permissible Limits in Foodstuffs

Several elements in tilapia muscle tissue exceeded international food safety thresholds and health‐based guidance values in the rainy season. Arsenic (inorganic), in particular, is of prominent concern, with estimated levels around 0.143 mg kg^−1^, substantially higher than the EU limit of 0.003 mg kg^−1^ but still within the maximum Brazilian ANVISA total limit of 1.0 mg kg^−1^. It is important to note that Brazilian regulations concerning this element are marked by interpretative and enforcement difficulties. The current regulation, RDC No. 722 (01/07/2022), establishes a maximum limit based on total As and requires arsenic speciation to assess inorganic As only when total concentrations exceed 1 mg kg^−1^ (wet weight). Compliance under this framework is evaluated using analytical methods that measure total As. In contrast, the Ministry of Agriculture, Livestock and Food Supply (MAPA) interprets the same threshold as applying exclusively to inorganic As, which represents the form of greatest toxicological concern. These differing regulatory interpretations have introduced uncertainty regarding how compliance should be determined and enforced for arsenic in food products.

Iron presented the highest absolute concentrations, reaching 7.01 mg kg^−1^, almost 9‐fold greater than the FDA limit of 0.75 mg kg^−1^. Although this element is essential for biological processes such as growth and cell proliferation, redox‐active iron compounds (Fe^2+^) can generate ROS, which can damage cellular functions and may lead to cell death [131]. Manganese was present at 0.144 mg kg^−1^, significantly surpassing the FDA's 0.1 mg kg^−1^ safety limit. This is an essential metal, acting as a cofactor in several enzymes important for brain function, such as glutamine synthetase and superoxide dismutase [132]. It can, however, impair metabolism when present at high levels, affecting carbohydrate processing and compromising the antioxidant system [133]. Nickel concentrations remained within the FDA limit of 0.04 mg kg^−1^, at 0.033 mg kg^−1^. Copper averaged 0.18 mg kg^−1^, only slightly lower than the recommended FDA maximum of 0.2 mg kg^−1^. Lead was detected at 0.016 mg kg^−1^, not exceeding both the EU threshold of 0.03 mg kg^−1^ and the FDA's 0.04 mg kg^−1^ limit. This element has the potential to trigger oxidative stress and harm cellular structures, thereby playing a role in the development of cancer [134]. Vanadium reached 0.04 mg kg^−1^, over 4‐fold the FDA limit of 0.01 mg kg^−1^, and Zn reached 5.35 mg kg^−1^, far exceeding the FDA's limit of 0.002 mg kg^−1^. Vanadium can disrupt metabolism and harm marine organisms' growth, reproduction, and survival, leading to ecological disturbances [135]. Zinc, essential for enzymatic functions, protein synthesis, and cellular metabolism, also acts as a cofactor for liver enzymes and contributes to MT synthesis [134]. At high levels, however, this element can interfere with the absorption and metabolism of other essential trace elements such as Cu and Fe, potentially leading to deficiencies and symptoms including nausea, vomiting, fever, headache, and abdominal pain [136].

Similarly to the rainy season, several elements exceeded international safety consumption thresholds during the dry season. Arsenic was consistently detected above the EU limit of 0.003 mg kg^−1^ w.w., at 0.19 mg kg^−1^ w.w., although still within the more lenient ANVISA limit of 1.0 mg kg^−1^ w.w. Nickel at 0.08 mg kg^−1^ w.w exceeded the FDA's recommended limit of 0.04 mg kg^−1^ w.w. Similarly, Cu was detected above the FDA's 0.2 mg kg^−1^ threshold across all sampled groups, with an average value of 0.28 mg kg^−1^ w.w. While this element is essential for combating anemia and sustaining metabolic processes, prolonged exposure to excessive levels can lead to liver and kidney damage, as well as the destruction of red blood cells [137]. Iron far exceeded the FDA's maximum limit of 0.75 mg kg^−1^ w.w. at 14.67 mg kg^−1^ w.w., suggesting excessive dietary intake of this element through tilapia consumption. Manganese (0.59 mg kg^−1^ w.w.), V (0.044 mg kg^−1^ w.w.), and Zn (6.92 mg kg^−1^ w.w.) were all present at levels exceeding FDA guidelines of 0.1 mg kg^−1^, 0.01 mg kg^−1^, and 0.002 mg kg^−1^ w.w., respectively.

The pattern of regulatory exceedances persisted when both dry and rainy seasons were analyzed together. Muscle As reached 0.17 mg kg^−1^ w.w., well above the EU limit, though still compliant with ANVISA's 1 mg kg^−1^ threshold. Nickel was found at 0.060 mg kg^−1^ w.w., significantly surpassing the FDA's 0.04 mg kg^−1^ w.w. limit. Similarly, Cu concentrations averaged 0.24 mg kg^−1^ w.w., slightly more than the FDA maximum of 0.2 mg kg^−1^. Iron reached an average of 11.28 mg kg^−1^ w.w., more than 15‐fold above the FDA's permissible value, suggesting a marked risk of overexposure. Manganese also remained high, with an average of 0.39 mg kg^−1^ w.w., exceeding 3‐fold the FDA limit. Zinc and V reached 6.22 mg kg^−1^ w.w. and 0.043 mg kg^−1^ w.w., respectively, both substantially higher than their FDA limits of 0.002 mg kg^−1^ and 0.01 mg kg^−1^ w.w., respectively.

It is important to note that no limits have been established for Ti in foodstuffs, to date, by any legislative organ. However, titanium dioxide (TiO_2_, E171) has been banned as a food additive in the European Union since 2022 due to genotoxicity and potential carcinogenicity concerns [138]. In the rainy season, its concentration was 6.53 mg kg^−1^ w.w. and increased to 8.15 mg kg^−1^ w.w. during the dry season, resulting in an average concentration of 7.43 mg kg^−1^ w.w. when grouping both seasons. Regular monitoring and research on the long‐term effects of Ti accumulation are paramount to understanding potential public health risks, given that this metal can bioaccumulate in aquatic organisms, although tested mostly in in vitro conditions [139].

#### 3.6.2 Estimated Daily and Weekly Intakes (EDI and EWI), Target Cancer Risk (TCR) and THQ Determinations

Across both rainy and dry seasons, EDI and EWI estimates (Tables S7 and S8) indicated that Ti and Zn were the elements that would be consumed in the highest absolute amounts. Zinc intake fell within the EU daily intake guidelines, which range from 7 to 25 mg kg^−1^ depending on the age group, and remained below the tolerable upper intake level for adults of 40 mg kg^−1^ day^−1^. For Fe, EDI and EWI values were calculated and compared with reference levels. The FAO/WHO recommends a tolerable upper intake level of 45 mg kg^−1^ day^−1^ for adults. The mean Fe intake between males and females was 0.005 mg kg^−1^ in the rainy season, 0.011 mg kg^−1^ in the dry season, and 0008 mg kg^−1^, all below the recommended doses. The EDI and EWI for Rb were higher in the dry season, whereas Co was lower in both periods. Iron and Zn intakes remained within established safety margins, while no reference values or regulatory limits currently exist for Rb and Ti.

Arsenic and Pb, both displaying the capacity to induce mutations, teratogenic effects, and carcinogenic outcomes in living organisms [20], were identified as the primary contributors to cancer risk, although TCR values (Tables S9–S12) for both remained within the acceptable range (10^−6^ < TCR < 10^−4^) even under the most heightened exposure frequency scenario, with no instances exceeding the threshold for unacceptable risk (TCR > 10^−4^) [140, 141] across all age groups, both sexes, and during dry, rainy, and combined seasons. Infants were found to be more susceptible, with female infants exhibiting the highest TCR value of 8.10 × 10^−8^.

Concerning the THQ (Tables S13–S17), values for As, Cu, Ni, V, and Zn were all below the safety threshold of 1 across all groups, with no significant increases observed even at the highest weekly intake frequency. Although these values remained below the threshold, Ni is recognized as a hazardous and potentially carcinogenic metal that can be substantially absorbed through dietary exposure [137], highlighting the importance of continuous monitoring. Similarly, the HI for all metals remained below 1, even under scenarios simulating consumption up to five times per week, indicating that cumulative non‐carcinogenic risks did not exceed established safety limits.

This integrated assessment emphasizes that chronic exposure risks may be underestimated, especially in human populations consuming substantial quantities of these metals through dietary sources, reinforcing the importance of ongoing monitoring and targeted public health interventions.

### 3.7 Antioxidant System

Superoxide dismutase activity is presented in Figure 5a, showing a significant 35.1% decrease in muscle during the dry period compared to the rainy period (maximum activity of 1.07 U mg^−1^ ptn, range: 0.451 to 2.41 U mg^−1^ ptn). The liver exhibited the opposite trend, with an 81.9% increase in SOD activity during the dry period compared to the rainy period. The lowest hepatic SOD activity was recorded during the rainy season (2.93 U mg^−1^ ptn, range: 0.93–5.56 U mg^−1^ ptn), while the lowest muscle activity was observed in the dry season (median: 0.694 U mg^−1^ ptn, range: 0.179–1.95 U mg^−1^ ptn). This enzyme plays a crucial role in the cellular defense against oxidative stress by catalyzing the dismutation of superoxide anions (O_2_^●−^) into hydrogen peroxide (H_2_O_2_), which is subsequently decomposed into water by CAT [117].

**FIGURE 5:**
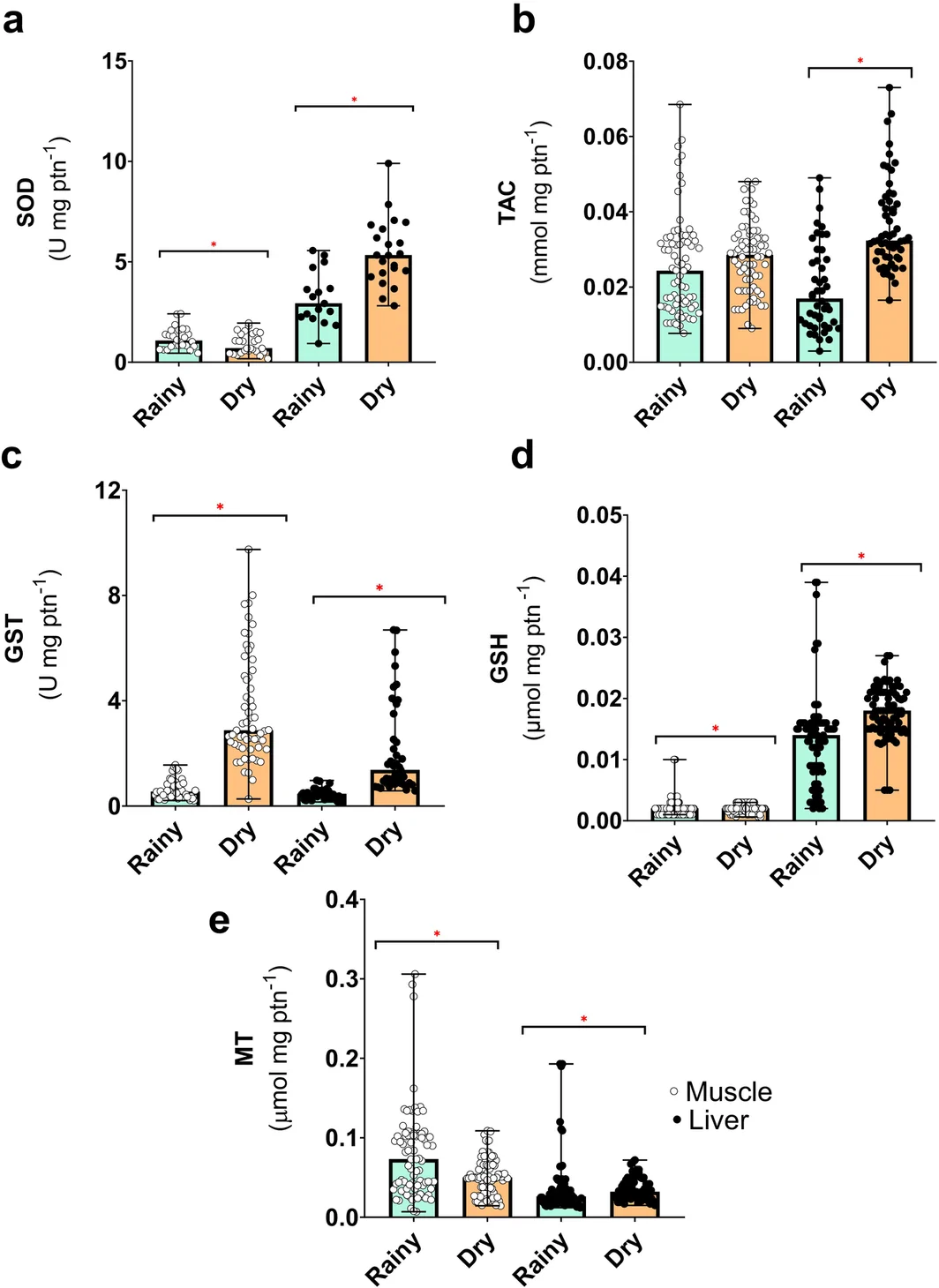
Antioxidant system responses in Nile tilapia ( *Oreochromis niloticus* ) muscle (○) and liver (●) from Piratininga Lagoon (PIR) during a rainy season—spring/summer—and dry season—autumn/winter. Biomarkers include: (a) superoxide dismutase (SOD), (b) total antioxidant capacity (TAC), (c) glutathione S‐transferase (GST), (d) reduced glutathione (GSH), and (e) metallothionein (MT). Data are presented as medians, with bounds indicating the maximum and minimum values. Asterisks (*) denote a significant seasonal comparison within the same tissue (Mann Whitney test, *p* < 0.05).

Glutathione S‐transferase is a key phase II detoxification enzyme that catalyzes the conjugation of electrophilic compounds with GSH, protecting cells from endogenous and exogenous toxicants [142]. A distinct GST behavior was observed in tilapia liver and muscle (Figure 5c), with significant differences in both tissues (*p* ≤ 0.05), decreasing significantly in both muscle and liver during the rainy season compared to the dry season (median: 0.547 U mg^−1^ ptn and 0.462 U mg^−1^ ptn, respectively). The observed reductions ranged from 81.2% in muscle to 70.4% in liver. Similar to GST, GSH levels showed significant seasonal variations in both muscle and liver tissues (Figure 5d). In muscle, GSH levels were 0.002 μmol mg^−1^ ptn during the rainy and dry seasons. The highest hepatic GSH levels were recorded during the dry season, representing a 28.6% increase compared to the rainy season. This tripeptide functions as a direct free radical scavenger and serves as a substrate cofactor for enzymes such as GPx and GST [31]. High GSH levels may reflect an adaptive cellular response to mitigate oxidative stress induced by chronic exposure to environmental contaminants [111].

Antioxidant enzymes alongside non‐enzymatic antioxidants, such as ascorbic acid, uric acid, citric acid, and vitamin E, act synergistically to protect cells against oxidative stress [143]. These compounds help neutralize ROS and interrupt free radical chain reactions [144]. The TAC measurement reflects this integrated response, providing an estimate of a tissue's ability to limit oxidative damage [145]. The dry season exhibited maximum TAC levels in both muscle and liver tissue (Figure 5b). However, a significant difference was detected only in liver samples (*p* ≤ 0.05), with a 14.7% decrease observed during the rainy period **in muscle to** 47.7% **in liver**.

Metallothioneins are low‐molecular‐weight, cysteine‐rich proteins that bind essential and non‐essential metals, playing a role in metal regulation and detoxification [107]. Their induction is widely used as a biomarker of metal exposure in both laboratory and field studies [146, 147]. Hepatic and muscle MT concentrations were significantly higher in rainy samples compared to the dry period (*p* ≤ 0.05) (Figure 5e). Muscle MT levels increased by 46%, while the increase in liver MT was low, at 23.1%. The lowest median muscle and liver MT concentrations were 0.050 μmol mg^−1^ ptn, range: 0.014–0.109 U mg^−1^ ptn, and 0.032 μmol mg^−1^ ptn, range: 0.015–0.072 U mg^−1^ ptn, respectively (*p* ≤ 0.05).

Metal and metalloid exposure is widely recognized as a major driver of oxidative stress in fish, primarily through the generation of ROS, disruption of redox homeostasis, and activation of enzymatic and non‐enzymatic antioxidant defenses [43, 130, 148]. The dry season was marked by more effective performance of the antioxidant system (SOD, GST, GSH, and TAC) in both liver and muscle tissues, possibly due to the higher toxic load recorded during this period, marked by high concentrations and greater diversity of elements in surface water. Such conditions appear to increase metabolic activity in fish tissues, particularly in the liver. The generation of ROS due to metal/metalloid accumulation is an inherent consequence of aerobic life [149]. To counteract oxidative damage, antioxidant enzymes such as SOD and CAT act as the first line of defense, while the glutathione system provides a secondary protective mechanism [34, 144]. Herein, the liver proved to be the most reactive organ during the dry season, corroborating its role as a primary site for detoxification and oxidative regulation. This pattern has been consistently reported in the literature, reinforcing the liver's importance in neutralizing ROS generated by metal exposure [150]. The coordinated activation of the first line (SOD) and second line (GST/GSH/MT) antioxidants suggests a systemic and integrated defense strategy, especially under conditions of elevated contamination [151, 152].

Several factors may influence metal accumulation in fish tissues. According to Moniruzzaman et al. [144], environmental metal concentrations, species‐specific traits, and feeding behavior are key determinants. For example, the gold‐spot mullet ( *Liza parsia* ), a pelagic omnivore similar to tilapia, tends to accumulate higher metal levels due to its capacity to feed across multiple water column strata, increasing its exposure to both dissolved and sediment‐bound contaminants [144]. This broader exposure may result in increased tissue damage and trigger antioxidant defenses. Additionally, other factors such as trophic level, food quality, age, and life history traits (e.g., growth, reproduction, and sex) can modulate both metal accumulation and oxidative responses [153, 154].

Regarding muscle tissue, although displaying a lower antioxidant response during the dry season, a marked increase in MT levels was observed during the rainy season, especially in this tissue, indicating probable bioaccumulation. Even in the context of reduced environmental metal concentrations in surface waters during the rainy season, muscle tissue maintained elevated MT levels. It suggests a possible redistribution or delayed detoxification process, in which metals in the MT‐enriched fraction are mobilized in response to physiological and environmental changes. Similar patterns have been noted under dietary Cd exposure, where, after 2 months of exposure plus 2 months of depuration, European seabass retained Cd mainly in detoxifying heat‐stable proteins/MT, whereas Senegalese sole sequestered Cd in organellar fractions and showed growth depression; evidence of redistribution and delayed detoxification via species‐specific pathways [155]. Other authors further emphasize that metals may remain compartmentalized in fish tissues [130], maintaining biological activity and toxicity through bioaccumulation, even when environmental levels decrease, highlighting the role of MT and antioxidant responses in chronic exposure scenarios.

Additionally, metal uptake and toxicity in aquatic organisms are influenced by various water physicochemical parameters, including pH, temperature, hardness, and salinity [154]. For instance, lower pH can increase metal solubility and ionization, while high hardness may reduce metal bioavailability [130]. High temperatures and salinity can alter metal speciation and complexation, often decreasing the availability of free ionic forms [156], which may further modulate detoxification responses and biomarker expression. Thus, the observed increase in MT levels does not necessarily indicate new exposure but may reflect a homeostatic adjustment to internal metal redistribution or long‐term contamination conditions [157, 158].

### 3.8 Oxidative Damage

Oxidative damage was assessed through LPO and PTC levels (Figure 6). Although no statistically significant differences were detected concerning MDA levels (*p* > 0.05), median values suggest opposite trends between tissues across seasons. During the dry period, MDA levels were 0.127 μmol mg^−1^ ptn (range: 0.051–0.486 μmol mg^−1^ ptn) in muscle and 0.930 μmol mg^−1^ ptn (range: 0.220–1.74 μmol mg^−1^ ptn) in liver. Lipid peroxidation is a classical oxidative stress indicator, resulting from an imbalance between ROS and cellular antioxidant systems [150], being MDA the most measured end‐product of polyunsaturated fatty acid oxidation, widely used as a cell membrane damage biomarker [159]. In one study, El‐Agri et al. [127] demonstrated that Fe and Cu can catalyze ROS generation and promote hydrogen abstraction from lipids, directly affecting membrane fluidity and cellular function. Similar results were reported by Moussa et al. [150] that found high metal levels and oxidative damage in the liver and gills of *C. gariepinus* and *O. niloticus* exposed to untreated discharges in Egypt. The absence of statistically significant differences in LPO may be related to high individual variability in fish responses. Nevertheless, the increase in MDA in the liver during the dry season may reflect the higher toxic burden in lagoons during this period, often linked to lower water volume and pollutant concentration. This pattern may also be related to the reproductive period, as observed by Inanan et al. [143], in which MDA levels increased during spawning, a time of high physiological demand and potential natural oxidative stress.

**FIGURE 6:**
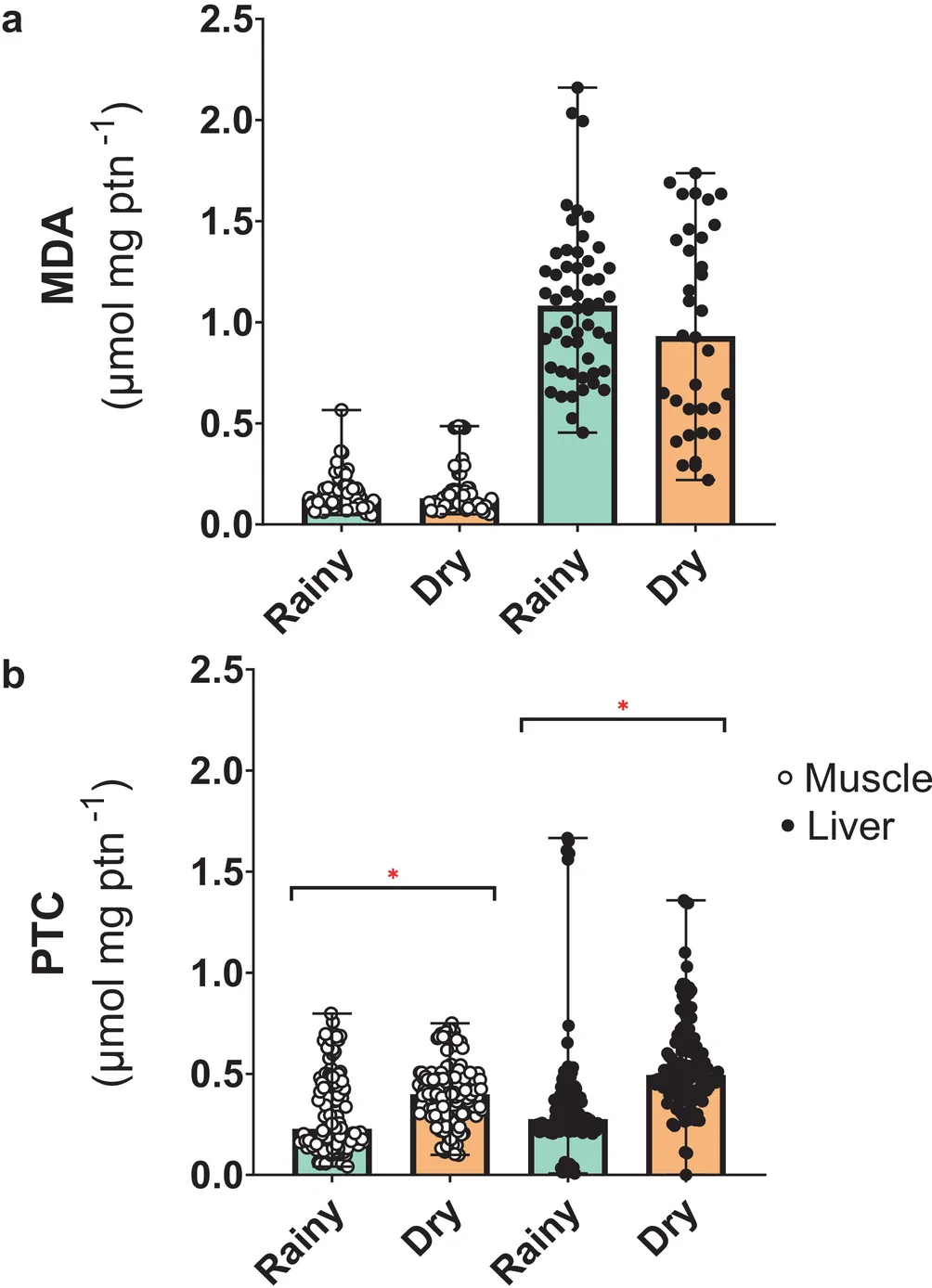
Oxidative damage in Nile tilapia ( *Oreochromis niloticus* ) muscle (○) and liver (●) from Piratininga Lagoon (PIR), Rio de Janeiro, southeastern Brazil, during rainy and dry seasons. Biomarkers include: (a) lipid peroxidation LPO, expressed as malondialdehyde (MDA) and (b) protein carbonylation (PTC). Data are presented as medians, with bounds indicating the maximum and minimum values. Asterisks (*) denote a significant seasonal difference within the same tissue (Mann–Whitney test, *p* < 0.05).

In contrast, PTC levels showed statistically significant differences between seasons (*p* ≤ 0.05), with more pronounced increases during the dry period in both muscle (75.7%) and liver (79.6%) compared to the rainy period (median: 0.226 and 0.274 μmol mg^−1^ ptn, respectively). Protein carbonylation is an irreversible modification induced by ROS that affects protein structure and function, considered an early and cumulative marker of oxidative stress [159]. The significant increase in PTC levels observed herein reinforces the hypothesis that the dry period imposed a greater oxidative challenge to organisms, likely due to a combination of metal pollution, pH fluctuations, and low oxygen availability. Previous studies have identified induced PTC in fish exposed to metal‐contaminated environments, particularly in the gills, liver, and muscle [25, 160, 161]. The presence of redox‐active metals, like Fe and Cu, favors Fenton reactions and the formation of highly reactive hydroxyl radicals, which directly attack amino acid side chains in proteins, promoting oxidation and carbonyl group formation [127]. Additionally, temperature and reproductive cycles also modulate oxidative stress responses [26]. During the dry season and spawning periods, ectothermic organisms such as fish may experience increased ROS generation due to higher oxygen availability in body fluids and decreased protein turnover, which can intensify oxidative damage, particularly in metabolically active tissues like liver [162, 163].

### 3.9 Correlations and PCA

Significant associations were observed between dissolved metals and metalloids in water and total concentrations in Nile tilapia muscle tissue (Figure 7), suggesting possible links between environmental exposure and tissue accumulation. These associations varied between seasons, likely reflecting changes in metal and metalloid bioavailability and uptake influenced by seasonal dynamics such as hydrological changes and pollutant mobilization. During the rainy season (Table S18), strong correlations were found among waterborne elements, such as As × Mg (*r* = 0.76), Fe × V (*r* = −0.78), and Mn × V (*r* = 0.90). Among total muscle concentrations, negative associations were noted between Pb × Ni (*r* = −0.74) and Mn × Ti (*r* = −0.71). Water As concentrations showed a negative correlation with muscle As levels (*r* = −0.61), potentially reflecting physiological regulation mechanisms such as detoxification, redistribution, or reduced uptake. Similar trends were observed in the dry season (Table S19), including strong intra‐water correlations (As × Mg, *r* = 0.75) and inverse associations in muscle (Rb × Ni and V × Zn, both *r* = −0.76). Additional cross‐element correlations were noted, such as water As × muscle Mo (*r* = −0.80) and water V × muscle Co (*r* = 0.55). This illustrates the complexity of metal and metalloid bioaccumulation, which may be modulated by species‐specific physiology, element interactions, and alternative exposure routes such as diet or sediment contact [28]. While elements like As, Pb, Ni, and V are known for their toxic potential, the accumulation of essential elements such as Zn and Mn, especially under environmental stress, may reflect oxidative imbalances or compensatory metabolic responses [130].

**FIGURE 7:**
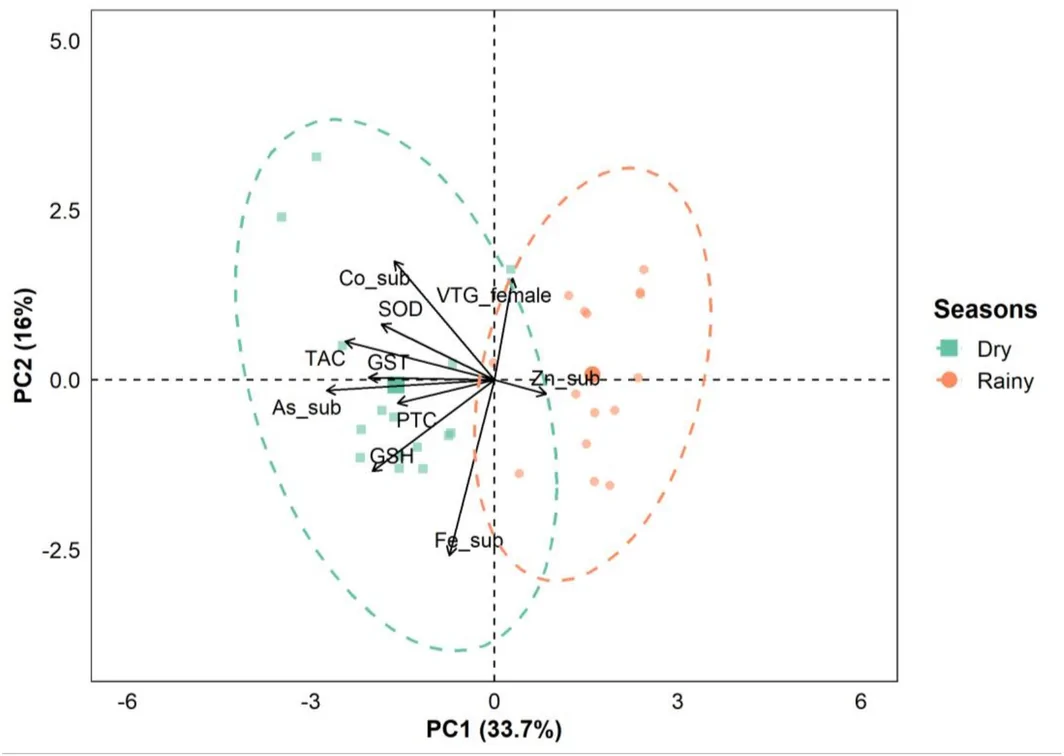
Principal component analysis (PCA) biplot based on biomarkers and subcellular metal and metalloid concentrations in Nile tilapia ( *Oreochromis niloticus* ) liver from Piratininga Lagoon (PIR), Rio de Janeiro, southeastern Brazil, during the dry (green squares) and rainy (orange circles) seasons. The analysis highlights seasonal separation along PC1 (33.7%) and PC2 (16%), with vectors representing the contribution and direction of each variable. Ellipses indicate 95% confidence intervals for each season.

The PCA was applied only to liver samples. Data suitability was verified by Bartlett's test of sphericity, the liver set met the assumptions (Bartlett's, *p* ≤ 0.05; KMO ≥ 0.5), whereas the muscle set did not (Bartlett's *p* > 0.05). The first two components explained 49.6% of the total variance, with PC1 (33.7%) capturing the main seasonal patterns. The PCA revealed a marked seasonal structuring of hepatic biochemical responses, with oxidative biomarkers and MT‐enriched subcellular fraction profiles shifting consistently between dry and rainy periods. Along with PC1, dry season samples (green squares) were clustered on the negative axis, while rainy season samples (orange dots) were concentrated on the positive side. This separation was driven mostly by GST (*r* = −0.67), SOD (*r* = −0.60), TAC, PTC, and subcellular As (*r* = −0.89), suggesting stronger antioxidant responses and potential metal‐induced stress under drier environmental conditions. MT‐enriched subcellular Co (*r* = −0.53) also contributed to PC1, suggesting involvement of Co partitioning in seasonal differentiation. In the same period, a significant sex effect in GSI and VTG ratio > 1 (male > female) was observed, suggestive of endocrine perturbation in males, yet strictly associative.

In contrast, MT‐enriched subcellular Fe (*r* = −0.84) and MT‐enriched subcellular Zn (*r* = 0.27) were associated with the rainy season. While MT‐enriched subcellular Zn exhibited low explanatory value (cos^2^ = 0.08), subcellular Fe was strongly represented in PC2 (cos^2^ = 0.71), indicating distinct MT‐enriched subcellular Fe dynamics during wet conditions, possibly linked to increased runoff or the remobilization of sediment‐bound metals [47, 164]. Vitellogenin in females showed moderate projection in both PC1 and PC2 and was aligned with dry season samples. This interpretation should, however, be treated with caution, as the high male–female VTG ratio (1.85) observed during the dry period suggests an atypical expression profile, possibly resulting from suppressed vitellogenin production in females or abnormal induction in males [37], possibly due to other EDCs.

Although correlation‐based analyses are valuable for identifying potential associations among metals and biological responses, they do not imply causality and should be interpreted with appropriate caution. This study was conducted under field‐based conditions in a complex urban lagoon, and the results therefore reflect environmentally realistic exposures influenced by natural biological variability and unmeasured environmental confounders, including potential interactions with co‐occurring pollutants. Fish samplings were restricted to a single representative site within the lagoon, which may not fully capture spatial heterogeneity across the system. However, this design allowed for controlled temporal (seasonal) comparisons while minimizing spatial variability as a confounding factor. Additionally, no ecologically comparable uncontaminated reference site was available for baseline comparison, a common limitation in highly urbanized coastal environments.

Sample sizes per sex and season were moderate, consistent with logistical constraints typical of ecological field studies. The absence of sediment metal and metalloid data also limited the evaluation of benthic exposure pathways for this demersal species, restricting a more comprehensive assessment of environmental compartments. Finally, the human health risk assessment relied on modeled fish consumption scenarios based on plausible local intake patterns for a single food item (tilapia), which may not fully represent dietary variability among different demographic groups. These considerations should be taken into account when extrapolating the findings. Nevertheless, they do not detract from the relevance of the integrated biochemical, subcellular, and physiological responses observed under chronic environmental exposure, nor from the value of this lagoon system as a model for assessing sublethal biological effects in urban coastal ecosystems.

## 4 Conclusion

Pronounced seasonal Nile tilapia contaminant exposure profiles and biological responses were observed herein. Despite exceedances of certain metals in muscle tissue, an integrated risk assessment indicates non‐carcinogenic and carcinogenic risks below safety thresholds, highlighting the importance of ongoing dietary exposure monitoring. Antioxidant biomarkers and metals and metalloids in the MT‐enriched subcellular fraction consistently distinguished dry and rainy periods, with the dry season marked by intensified oxidative stress (SOD, GSH, GST, and TAC) and altered metal and metalloid profiles, including increased hepatic and muscle As, Co, Fe, Rb, and Se levels. Additionally, the detection of an inverted male/female VTG ratio during the dry period points to potential endocrine disruption, possibly triggered by exposure to diverse EDCs. Significant negative correlations between water and intracellular metals and metalloids (e.g., As) suggest complex physiological regulation involving detoxification, redistribution, or altered metal uptake. The bioaccumulation of essential metals like Zn and Mn, as well as toxic elements such as As, Pb, Ni, and V, may reflect oxidative imbalance or compensatory metabolic processes under stress conditions.

These results reinforce the relevance of seasonal monitoring and multi‐biomarker approaches in urban coastal environments. Future long‐term monitoring programs are recommended to determine whether the observed seasonal patterns represent persistent trends or short‐term fluctuations influenced by broader environmental drivers, such as climate variability or land‐use changes. Expanding spatial coverage and incorporating additional environmental compartments may further refine the understanding of contamination dynamics. Moreover, future risk assessments may adjust exposure parameters to better represent specific demographic groups, including children, subsistence fishers, or high‐frequency consumers, thereby improving the precision of human health risk characterization for vulnerable populations.

## Author Contributions

**Julia Araújo Alves:** investigation, formal analysis, data curation, writing – original draft, visualization. **Priscila M. O. Muniz Cunha:** methodology, data curation. **Shênia Patrícia Corrêa Novo:** investigation, formal analysis. **Lorena Oliveira Souza Soares:** data curation. **Tatiana Dillenburg Saint‐Pierre:** methodology, data curation, writing – review and editing. **Fábio Veríssimo Correia:** conceptualization, methodology, supervision. **André Luís de Sá Salomão:** methodology, formal analysis, supervision, validation, writing – review and editing. **Rachel Ann Hauser‐Davis:** conceptualization, methodology, funding acquisition, writing – review and editing. **Enrico Mendes Saggioro:** conceptualization, methodology, supervision, project administration, funding acquisition, writing – review and editing.

## Funding

This work was supported by Fundação Carlos Chagas Filho de Amparo à Pesquisa do Estado do Rio de Janeiro (E‐26/204.405/2024, E‐26/211.268/2021, E‐26/21.460/2019), National Council for Scientific and Technological Development (CNPq) (303248/2024‐6), and Coordination for the Improvement of Higher Education Personnel (CAPES) (001/2021).

## Supporting information


**Table S1:** Limits of detection (LOD) and limits of quantification (LOQ) (mg kg^−1^) of metal and metalloid concentrations in tilapia tissues ( *Oreochromis niloticus* ) and surface water (mg L^−1^) from Piratininga Lagoon, Rio de Janeiro, southeastern Brazil. Values are expressed as mean ± standard deviation.
**Table S2:** Observed and certified values (mg kg^−1^) and recovery percentages for the metals and metalloids present in the Certified Reference Material (DORM‐5, National Research Council Canada).
**Table S3:** Physicochemical determinations in water from five sampling campaigns conducted at Piratininga Lagoon, Rio de Janeiro, southeastern Brazil. NA = not applicable. pH = potential of hydrogen; DO = dissolved oxygen; EC = electrical conductivity; Turb. = turbidity; Temp. = temperature; ORP = oxidation–reduction potential; TDS = total dissolved solids; COD = chemical oxygen demand; BOD_5_ = biochemical oxygen demand; *P* = total phosphorus; TN = total nitrogen; CtT = thermotolerant coliforms; *E. coli* = *Escherichia coli* . ^a^BOD estimated from the BOD/COD ratio, with a value of 0.5 for the sampling points 165.
**Table S4:** Metal and metalloid concentrations in surface water (mg L^−1^) from Piratininga Lagoon, Rio de Janeiro, southeastern Brazil. Values are expressed as mean ± standard deviation. < LOQ: values lower than the Limit of Quantification.
**Table S5:** Biometric data (mean ± standard deviations) of *Oreochromis niloticus* during the dry (winter and autumn) and rainy (spring and summer) seasons from Piratininga Lagoon, Rio de Janeiro, Brazil.
**Table S6:** Total metal and metalloid concentrations (mg kg^−1^ w.w.) in Nile tilapia ( *Oreochromis niloticus* ) muscle from Piratininga Lagoon, Rio de Janeiro Brazil. Results are expressed as median values with minimum and maximum values. < LOQ indicates samples below the Limit of Quantification.
**Table S7:** Estimated daily intake (EDI) calculations concerning *Oreochromis niloticus* consumption by adult, adolescent, child, and infant males and females during the dry, rainy, and mixed seasons.
**Table S8:** Estimated weekly intake (EWI) calculations concerning *Oreochromis niloticus* consumption by adult, adolescent, child, and infant males and females during the dry, rainy, and mixed seasons.
**Table S9:** Target cancer risk (TCR) calculated for As from consumption of *Oreochromis niloticus* across age groups, consumption frequencies (including once per week), and dry, rainy, and mixed seasons. No values exceeded legal limits of 10^−6^ < TCR < 10^−4^, indicating acceptable risk, and TCR > 10^−4^, indicating unacceptable risk.
**Table S10:** Target cancer risk (TCR) calculated for 1% As from consumption of *Oreochromis niloticus* across age groups, consumption frequencies (including once per week), and dry, rainy, and mixed seasons. No values exceeded legal limits of 10^−6^ < TCR < 10^−4^, indicating acceptable risk, and TCR > 10^−4^, indicating unacceptable risk.
**Table S11:** Target cancer risk (TCR) calculated for 10% As from consumption of *Oreochromis niloticus* across age groups, consumption frequencies (including once per week), and dry, rainy, and mixed seasons. No values exceeded legal limits of 10^−6^ < TCR < 10^−4^, indicating acceptable risk, and TCR > 10^−4^, indicating unacceptable risk.
**Table S12:** Target cancer risk (TCR) calculated for Pb from consumption of *Oreochromis niloticus* across age groups. Consumption frequencies of 1–5 times per week and dry, rainy, and mixed seasons. No values exceeded legal limits of 10^−6^ < TCR < 10^−4^, indicating acceptable risk, and TCR > 10^−4^, indicating unacceptable risk.
**Table S13:** Target hazard quotient (THQ) values for As, Cu, Ni, V and Zn as well as the Hazard Index (HI) calculated from the ingestion of *Oreochromis niloticus* one time a week by male and female infants, children, and adults on dry, rainy, and mixed seasons. No values exceeded legal limits of THQ < 1 indicating no carcinogenic effects expected and THQ > 1 indicating the possibility of adverse health effects.
**Table S14:** Target hazard quotient (THQ) values for s, Cu, Ni, V and Zn as well as the Hazard Index (HI) calculated from the ingestion of *Oreochromis niloticus* two times a week by male and female infants, children, and adults on dry, rainy, and mixed seasons. No values exceeded legal limits of THQ < 1 indicating no carcinogenic effects expected and THQ > 1 indicating the possibility of adverse health effects.
**Table S15:** Target hazard quotient (THQ) values for As, Cu, Ni, V and Zn as well as the Hazard Index (HI) calculated from the ingestion of *Oreochromis niloticus* three times a week by male and female infants, children and adults during the dry, rainy, and grouped seasons. No values exceeded legal limits of THQ < 1 indicating no carcinogenic effects expected and THQ > 1 indicating the possibility of adverse health effects.
**Table S16:** Target hazard quotient (THQ) values for As, Cu, Ni, V and Zn as well as the Hazard Index (HI) calculated from the ingestion of *Oreochromis niloticus* four times a week by male and female infants, children and adults during the dry, rainy, and grouped seasons. No values exceeded legal limits of THQ < 1 indicating no carcinogenic effects expected and THQ > 1 indicating the possibility of adverse health effects.
**Table S17:** Target hazard quotient (THQ) values for As, Cu, Ni, V and Zn as well as the Hazard Index (HI) calculated from the ingestion of *Oreochromis niloticus* five times a week by male and female infants, children and adults during the dry, rainy, and grouped seasons. No values exceeded legal limits of THQ < 1 indicating no carcinogenic effects expected and THQ > 1 indicating the possibility of adverse health effects.
**Table S18:** Significant Spearman correlation matrices between dissolved metals in surface water (suffix: _w) and total metal (suffix: _t) concentrations, during rainy season in Nile tilapia ( *Oreochromis niloticus* ) muscle samples. Only statistically significant correlations (*p* < 0.05) are shown.
**Table S19:** Significant Spearman correlation matrices between dissolved metals in surface water (suffix: _w) and total metal (suffix: _t) concentrations, during dry season in Nile tilapia ( *Oreochromis niloticus* ) muscle samples. Only statistically significant correlations (*p* < 0.05) are shown.

## Data Availability

The data that support the findings of this study are available on request from the corresponding author. The data are not publicly available due to privacy or ethical restrictions.
