# Supplementary material for: Assessment of Oxidative Stress, Vitellogenin, and Human Health Risks Derived From Nile Tilapia Exposed to Metals and Metalloids in Southeastern Brazil
Source: Environ Toxicol. 2026 Apr 15;41(12):1701–25. doi: 10.1002/tox.70094 (PMC13749271; doi:10.1002/tox.70094)
Supplement: Supplementary file 1 [file TOX-41-1701-s001.docx]

**Assessment of Oxidative Stress, Vitellogenin, and Human Health Risks in Nile Tilapia Exposed to Metals in Southeastern Brazil**

Julia Araújo Alves^1^, Priscila M. O. Muniz Cunha^2^, Shênia Patrícia Corrêa Novo^3^, Lorena Oliveira Souza Soares^1^, Tatiana Dillenburg Saint-Pierre^4^, Fábio Veríssimo Correia ^5^; André Luís de Sá Salomão^2^; Rachel Ann Hauser-Davis ^1^, Enrico Mendes Saggioro^1^.

^1^ Environmental Health Assessment and Promotion Laboratory (LAPSA), Oswaldo Cruz Institute (IOC), Oswaldo Cruz Foundation (Fiocruz), Rio de Janeiro, Brazil.

^2^Bioremediation and Phytotechnology Laboratory (LABIFI), Department of Environmental Engineering, Rio de Janeiro State University (UERJ), Rio de Janeiro, Brazil.

3 Laboratory of Comparative and Environmental Virology (LVCA), Oswaldo Cruz Institute (IOC), Oswaldo Cruz Foundation (Fiocruz), Rio de Janeiro, Brazil.

^4^Atomic Spectrometry Laboratory (LABSPECTRO), Department of Chemistry, Pontifical Catholic University of Rio de Janeiro (PUC-Rio), Rio de Janeiro, Brazil.

^5^Environmental Health Laboratory, Department of Natural Sciences, Federal University of the State of Rio de Janeiro (UNIRIO), Rio de Janeiro, Brazil.

**Table S1:** Limits of Detection (LOD) and Limits of Quantification (LOQ) (mg kg^–1^) of metal and metalloid concentrations in tilapia tissues (Oreochromis niloticus) and surface water (mg L^–1^) from Piratininga Lagoon, Rio de Janeiro, Southeastern Brazil. Values are expressed as means ± standard deviations.

| element | LOD | | | LOQ | | |
| --- | --- | --- | --- | --- | --- | --- |
|  | Water (mg L^–1^) | Total metals (mg kg^–1^) | Subcellular metals (mg kg^–1^) | Water (mg L^–1^) | Total metals (mg kg^–1^) | Subcellular metals (mg kg^–1^) |
| As | 0.009 | 0.002 | 0.004 | 0.029 | 0.007 | 0.014 |
| Co | 0.0001 | 0.0001 | 0.001 | 0.0003 | 0.001 | 0.002 |
| Cu | 0.006 | 0.007 | 0.012 | 0.020 | 0.022 | 0.040 |
| Fe | 0.61 | 0.901 | 1.049 | 2.00 | 3.004 | 3.500 |
| Hg | 0.003 | 0.003 | 0.002 | 0.010 | 0.011 | 0.008 |
| Mn | 0.004 | 0.006 | 0.002 | 0.010 | 0.021 | 0.010 |
| Mo | 0.005 | 0.0001 | 0.009 | 0.017 | 0.001 | 0.030 |
| Ni | 0.004 | 0.002 | 0.007 | 0.010 | 0.007 | 0.024 |
| Pb | 0.002 | 0.001 | 0.003 | 0.007 | 0.003 | 0.009 |
| Rb | 0.001 | 0.001 | 0.001 | 0.002 | 0.002 | 0.002 |
| Se | 0.01 | 0.098 | 0.076 | 0.05 | 0.326 | 0.250 |
| Ti | 0.02 | 0.052 | 0.009 | 0.07 | 0.173 | 0.030 |
| V | 0.01 | 0.0001 | 0.002 | 0.04 | 0.001 | 0.007 |
| Zn | 0.03 | 0.005 | 0.122 | 0.11 | 0.018 | 0.405 |

**Table S2:** Observed and certified values (mg kg^-1^) and recovery percentages for the Certified Reference Material (DORM-5, National Research Council Canada).

|  | DORM-5 | | |
| --- | --- | --- | --- |
| Elements | Observed (%) | Certified  (%) | Recovery (%) |
| As | 14.4 | 13.3 | 108.9 |
| Co | 0.04 | 0.06 | 71.54 |
| Cd | 0.167 | 0.148 | 112.88 |
| Cu | 2.79 | 3.30 | 84.71 |
| Fe | 79.34 | 113.00 | 70.21 |
| Hg | 0.35 | 0.31 | 117.25 |
| Mn | 0.76 | 1.06 | 71.65 |
| Mo | 0.12 | 0.13 | 98.08 |
| Ni | 0.35 | 0.44 | 94.76 |
| Pb | 0.06 | 0.05 | 117.66 |
| Rb | 2.19 | 2.76 | 79.53 |
| Se | 2.91 | 2.40 | 108.74 |
| V | 0.396 | 0.347 | 110.02 |
| Zn | 33.77 | 28.70 | 117.67 |

**Table S3:** Physicochemical determinations in water from five sampling campaigns conducted at Piratininga Lagoon, Rio de Janeiro, southeastern Brazil. NA= not applicable. pH = potential of hydrogen; DO = dissolved oxygen; eC = electrical conductivity; Turb. = turbidity; Temp. = temperature; ORP = oxidation–reduction potential; TDS = total dissolved solids; COD = chemical oxygen demand; BOD₅ = biochemical oxygen demand; P = total phosphorus; TN = total nitrogen; CtT = thermotolerant coliforms; E. coli = escherichia coli. ^a^ BOD estimated from the BOD/COD ratio, with a value of 0.5 for the sampling points (Bollmann e Marques, 2006).

| Sample Point | | pH | DO (mg L^-1^) | DO (%) | eC (mS/cm²) | Turb. (NTU) | Temp. (ºC) | ORP (mV) | TDS (g L^-1^) | Salinity (PSU) | COD (mg L^-1^) | BOD ^a^  (mg L^-1^) | P  (mg L^-1^) | TN  (mg L^-1^) | CtT (NMP/ 100mL) | *E. coli (*NMP/100mL) |
| --- | --- | --- | --- | --- | --- | --- | --- | --- | --- | --- | --- | --- | --- | --- | --- | --- |
| Dry season 1  (winter) | **P1** | 9.9 | 11.7 | 165.0 | 33.2 | 43.8 | 22.7 | 7.5 | NA | 21.4 | 114.7 | 22.9 | NA | 1.8 | 3410000.0 | 1000.0 |
|  | **P2** | 9.7 | 10.4 | 128.6 | 30.7 | 30.2 | 20.0 | 11.7 | NA | 21.3 | 117.4 | 23.5 | NA | 1.4 | 31800.0 | NA |
|  | **P3** | 9.5 | 11.7 | 143.6 | 29.8 | 31.5 | 20.8 | 14.2 | NA | 20.3 | 140.2 | 28.0 | NA | NA | 9300.0 | NA |
|  | **P4** | 9.6 | 11.6 | 144.7 | 16.3 | 31.8 | 20.5 | 13.8 | NA | 20.5 | 111.9 | 22.4 | NA | NA | 7500.0 | NA |
|  | **P5** | 9.8 | 8.1 | 124.8 | 33.8 | 38.6 | 22.7 | 10.3 | NA | 21.3 | 90.2 | 18.0 | NA | NA | 35000.0 | NA |
| Rainy season 1 (spring | **P1** | 9.0 | 7.2 | 101.3 | 31.5 | 12.4 | 28.0 | 8.8 | 1.493 | 18.4 | 95.2 | 19.0 | NA | 3.1 | 104620.0 | NA |
|  | **P2** | 9.1 | 8.5 | 121.3 | 30.7 | 13.7 | 27.9 | 9.0 | 14.500 | 17.9 | 105.2 | 21.0 | NA | 4.1 | 65860.0 | 2000.0 |
|  | **P3** | 9.0 | 5.3 | 80.9 | 28.8 | 19.9 | 31.9 | -228.0 | 15.100 | 17.2 | 107.4 | 21.5 | NA | 4.2 | 68670.0 | NA |
|  | **P4** | 9.1 | 5.1 | 75.7 | 29.2 | 10.7 | 32.5 | -36.0 | 14.900 | 17.9 | 111.9 | 22.4 | NA | 3.2 | 38730.0 | 5100.0 |
|  | **P5** | 8.9 | 7.1 | 99.9 | 32.3 | 15.9 | 27.5 | 9.1 | 15.400 | 19.1 | 85.8 | 17.2 | NA | 3.4 | 46110.0 | 2000.0 |
| Rainy season 2 (summer) | **P1** | 9.0 | 7.2 | 99.1 | 1740 | 47.3 | 28.8 | 29.0 | 10.900 | 10.3 | 130.5 | 26.1 | 0.8 | 4.1 | 2419.6 | 100 |
|  | **P2** | 9.1 | 7.7 | 107.2 | 1700 | 44.5 | 29.4 | 36.0 | 10.500 | 10.0 | 883.8 | 176.8 | 0.8 | 14.7 | 2419.6 | 410 |
|  | **P3** | 9.1 | 6.5 | 88.5 | 1560 | 65.8 | 31.7 | 26.0 | 9.820 | 9.0 | 92.2 | 18.4 | 1.3 | 32.0 | 2419.6 | 100 |
|  | **P4** | 9.2 | 7.6 | 110.3 | 1650 | 57.1 | 32.8 | 26.0 | 10.200 | 9.6 | 132.2 | 26.4 | 1.1 | 4.1 | 2419.6 | 100 |
|  | **P5** | 8.9 | 10.0 | 138.1 | 1900 | 35.2 | 28.5 | 55.0 | 11.800 | 11.3 | 158.0 | 31.6 | 0.4 | 3.6 | 2419.6 | 100 |
| Dry season 2  (autumn) | **P1** | 8.2 | 8.0 | 99.2 | 26 | 17.6 | 21.3 | 0.0 | 161.000 | 15.9 | 118.8 | 23.8 | NA | 2.72 | 91390.0 | 4140.0 |
|  | **P2** | 8.5 | 12.7 | 154.6 | 25.2 | 17.2 | 20.7 | 43.0 | 156.000 | 15.3 | 94.7 | 18.9 | NA | NA | NA | 3100.0 |
|  | **P3** | 8.7 | 9.7 | 130.5 | 244 | 31.5 | 24.1 | -50.0 | 152.000 | 14.8 | 96.3 | 19.3 | 0.7 | NA | 141360.0 | 6140.0 |
|  | **P4** | 8.5 | 7.6 | 99.3 | 239 | 26.4 | 24.5 | -35.0 | 148.000 | 14.5 | 108.0 | 21.6 | NA | 3.01 | 241960.0 | 4090.0 |
|  | **P5** | 8.4 | 12.1 | 154.0 | 28 | 29.90 | 22.4 | 229.0 | 174.000 | 17.2 | 87.2 | 17.4 | 0.6 | 2.24 | 96060.0 | 3210.0 |
| Limits (CONAMA 357/2005, Class 2, brackish waters) ^b^Maximum in up to 20% of samples (minimum of 6 bimonthly samples within one year).” | | 6.5-8.5 | ≥0.4 | NA | NA | NA | NA | NA | NA | NA | NA | NA | 0.186 | 0.70 | <2500^b^ | NA |

**Table S4:** Metal and metalloid concentrations in surface water (mg L^–1^) from Piratininga Lagoon, Rio de Janeiro, Southeastern Brazil. Values are expressed as means ± standard deviations (SD). <LOQ - values lower than the Limit of Quantification.

|  | **Rainy season** | **Dry season** |
| --- | --- | --- |
|  | Mean ± SD  (min-max) | Mean ± SD  (min-max) |
| **As** | 0.074 ±0.026  (0.030-0.090) | 0.057 ± 0.004  (0.053-0.062) |
| **Fe** | < LOQ | 1.20 ± 0.13  (1.06-1.45) |
| **Hg** | < LOQ | 0.006 ± 0.001  (0.005-0.007) |
| **Mg** | 432.8 ± 364.6  (61-903) | 522.5 ± 26.9  (494.1-572.5) |
| **Mn** | 0.076 ± 0.042  (0.040-0.140) | 0.036 ± 0.003  (0.032-0.043) |
| **Mo** | 0.009 ± 0.002  (0.005-0.011) | 0.071 ± 0.066  (0.024-0.183) |
| **Ni** | < LOQ | 0.005 ± 0.001  (0.004-0.007) |
| **Pb** | < LOQ | 0.002±0,002  (0.001-0.006) |
| **Rb** | 0.050 ± 0.039  (0.009-0.102) | 0.0701± 0.004  (0.063-0.075) |
| **Ti** | 0.163 ± 0.035  (0.120-0.200) | 0.025 ± 0.011  (0.013-0.049) |
| **V** | 0.045 ± 0.017  (0.017-0.059) | 0.070 ± 0.010  (0.060-0.087) |
| **Zn** | < LOQ | 0.121 ± 0.005  (0.114-0.134) |

**Table S5:** Biometric data (means ± standard deviations) of Oreochromis niloticus during the dry (winter and autumn) and rainy (spring and summer) seasons from Piratininga Lagoon, Rio de Janeiro, Brazil.

|  | | Dry season | Rainy season |
| --- | --- | --- | --- |
|  | | mean ± SD  (min-max) | mean ± SD  (min-max) |
| Length (cm) | | 258.0 ± 142.8  (64.66 – 599.3) | 362.9 ± 220.7  (134.1 – 1040) |
| Weight (g) | | 22.45 ± 4.68  (14.5 – 31.0) | 25.7 ± 4.82  (19 – 39) |
| Sex | Female | 13 | 14 |
|  | Male | 16 | 11 |

**Table S6:** Total metal and metalloid concentrations (mg kg^–1^ w.w.) in Nile tilapia (Oreochromis niloticus) muscle from Piratininga Lagoon. Rio de Janeiro. Brazil. Results are expressed as median values with minimum and maximum values. <LOQ indicates samples below the Limit of Quantification.

|  | Dry season  mg kg^-1^ (w.w.) | Rainy season  mg kg^-1^ (w.w.) |
| --- | --- | --- |
|  | Median  (min-max) | Median  (min-max) |
| As | 0.191  (0.102-0.301) | 0.118  (0.072-0.282) |
| Co | 0.014  (0.002-0.017) | 0.005  (0.003-0.010) |
| Cu | 0.231  (0.138-0.536) | 0.174  (0.110-0.300) |
| Fe | 6.72  (3.48-11.3) | 6.64  (4.78-11.02) |
| Mn | 0.234  (0.122-0.539) | 0.133  (0.077-0.276) |
| Mo | 0.002  (0.001-0.005) | <LOQ |
| Ni | 0.028  (0.007-0.081) | 0.024  (0.011-0.063) |
| Pb | 0.010  (0.004-0.031) | 0.008  (0.004-0.015) |
| Rb | 2.43  (1.747-3.800) | 2.17  (1.71-3.15) |
| Ti | 6.30  (5.26-10.04) | 6.47  (4.81-9.12) |
| V | 0.029  (0.020-0.0510) | 0.041  (0.029-0.064) |
| Zn | 6.28  (4.085-12.6) | 4.58  (3.05-8.35) |

**Table S7:**Estimated Daily Intake (EDI) calculations concerning Oreochromis niloticus consumption by adult, adolescent, child, and infant males and females during the dry, rainy, and mixed seasons.

|  | **Group** | **Sex** | **As** | **Co** | **Cu** | **Fe** | **Mn** | **Mo** | **Ni** | **Pb** | **Rb** | **Ti** | **V** | **Zn** |
| --- | --- | --- | --- | --- | --- | --- | --- | --- | --- | --- | --- | --- | --- | --- |
| **EDI Rainy Season** | Babies | Males | 1.95e-04 ± 7.82e-05 | 7.59e-06 ± 2.90e-06 | 2.48e-04 ± 7.59e-05 | 9.56e-03 ± 2.41e-03 | 1.96e-04 ± 7.40e-05 | NA | 4.51e-05 ± 3.04e-05 | 2.13e-05 ± 2.24e-05 | 3.05e-03 ± 5.18e-04 | 8.90e-03 ± 1.29e-03 | 5.82e-05 ± 1.17e-05 | 7.29e-03 ± 2.64e-03 |
|  |  | Females | 2.14e-04 ± 8.60e-05 | 8.35e-06 ± 3.19e-06 | 2.73e-04 ± 8.35e-05 | 1.05e-02 ± 2.65e-03 | 2.16e-04 ± 8.14e-05 | NA | 4.96e-05 ± 3.35e-05 | 2.35e-05 ± 2.47e-05 | 3.36e-03 ± 5.70e-04 | 9.80e-03 ± 1.41e-03 | 6.40e-05 ± 1.29e-05 | 8.02e-03 ± 2.91e-03 |
|  | Children | Males | 1.49e-04 ± 5.97e-05 | 5.80e-06 ± 2.22e-06 | 1.90e-04 ± 5.80e-05 | 7.31e-03 ± 1.84e-03 | 1.50e-04 ± 5.65e-05 | NA | 3.44e-05 ± 2.32e-05 | 1.63e-05 ± 1.71e-05 | 2.33e-03 ± 3.96e-04 | 6.80e-03 ± 9.82e-04 | 4.44e-05 ± 8.93e-06 | 5.57e-03 ± 2.02e-03 |
|  |  | Females | 1.62e-04 ± 6.52e-05 | 6.32e-06 ± 2.42e-06 | 2.07e-04 ± 6.32e-05 | 7.97e-03 ± 2.01e-03 | 1.64e-04 ± 6.16e-05 | NA | 3.75e-05 ± 2.54e-05 | 1.78e-05 ± 1.87e-05 | 2.54e-03 ± 4.32e-04 | 7.42e-03 ± 1.07e-03 | 4.85e-05 ± 9.74e-06 | 6.08e-03 ± 2.20e-03 |
|  | Adults | Males | 1.02e-04 ± 4.10e-05 | 3.98e-06 ± 1.52e-06 | 1.30e-04 ± 3.98e-05 | 5.01e-03 ± 1.26e-03 | 1.03e-04 ± 3.87e-05 | NA | 2.36e-05 ± 1.59e-05 | 1.12e-05 ± 1.17e-05 | 1.60e-03 ± 2.71e-04 | 4.66e-03 ± 6.73e-04 | 3.05e-05 ± 6.12e-06 | 3.82e-03 ± 1.38e-03 |
|  |  | Females | 1.07e-04 ± 4.30e-05 | 4.17e-06 ± 1.60e-06 | 1.36e-04 ± 4.17e-05 | 5.26e-03 ± 1.33e-03 | 1.08e-04 ± 4.07e-05 | NA | 2.48e-05 ± 1.67e-05 | 1.17e-05 ± 1.23e-05 | 1.68e-03 ± 2.85e-04 | 4.90e-03 ± 7.07e-04 | 3.20e-05 ± 6.43e-06 | 4.01e-03 ± 1.45e-03 |
| **EDI Dry Season** | Babies | Males | 2.58e-04 ± 7.23e-05 | 1.94e-05 ± 2.60e-05 | 3.81e-04 ± 2.04e-04 | 2.00e-02 ± 2.55e-02 | 8.08e-04 ± 1.75e-03 | 3.31e-06 ± 1.67e-06 | 1.12e-04 ± 2.42e-04 | 2.76e-05 ± 4.15e-05 | 3.27e-03 ± 5.89e-04 | 1.11e-02 ± 7.36e-03 | 5.97e-05 ± 4.79e-05 | 9.44e-03 ± 3.22e-03 |
|  |  | Females | 2.84e-04 ± 7.95e-05 | 2.14e-05 ± 2.86e-05 | 4.19e-04 ± 2.24e-04 | 2.20e-02 ± 2.81e-02 | 8.89e-04 ± 1.92e-03 | 3.64e-06 ± 1.83e-06 | 1.23e-04 ± 2.66e-04 | 3.03e-05 ± 4.57e-05 | 3.60e-03 ± 6.48e-04 | 1.22e-02 ± 8.10e-03 | 6.57e-05 ± 5.27e-05 | 1.04e-02 ± 3.55e-03 |
|  | Children | Males | 1.98e-04 ± 5.52e-05 | 1.48e-05 ± 1.99e-05 | 2.91e-04 ± 1.56e-04 | 1.53e-02 ± 1.95e-02 | 6.17e-04 ± 1.33e-03 | 2.53e-06 ± 1.27e-06 | 8.53e-05 ± 1.85e-04 | 2.11e-05 ± 3.17e-05 | 2.50e-03 ± 4.50e-04 | 8.49e-03 ± 5.62e-03 | 4.56e-05 ± 3.66e-05 | 7.21e-03 ± 2.46e-03 |
|  |  | Females | 2.15e-04 ± 6.03e-05 | 1.62e-05 ± 2.17e-05 | 3.18e-04 ± 1.70e-04 | 1.67e-02 ± 2.13e-02 | 6.73e-04 ± 1.45e-03 | 2.76e-06 ± 1.39e-06 | 9.31e-05 ± 2.02e-04 | 2.30e-05 ± 3.46e-05 | 2.73e-03 ± 4.91e-04 | 9.26e-03 ± 6.13e-03 | 4.98e-05 ± 3.99e-05 | 7.87e-03 ± 2.69e-03 |
|  | Adults | Males | 1.35e-04 ± 3.79e-05 | 1.02e-05 ± 1.36e-05 | 2.00e-04 ± 1.07e-04 | 1.05e-02 ± 1.34e-02 | 4.23e-04 ± 9.14e-04 | 1.73e-06 ± 8.73e-07 | 5.85e-05 ± 1.27e-04 | 1.44e-05 ± 2.17e-05 | 1.71e-03 ± 3.09e-04 | 5.82e-03 ± 3.86e-03 | 3.13e-05 ± 2.51e-05 | 4.94e-03 ± 1.69e-03 |
|  |  | Females | 1.42e-04 ± 3.98e-05 | 1.07e-05 ± 1.43e-05 | 2.10e-04 ± 1.12e-04 | 1.10e-02 ± 1.40e-02 | 4.44e-04 ± 9.60e-04 | 1.82e-06 ± 9.17e-07 | 6.14e-05 ± 1.33e-04 | 1.52e-05 ± 2.28e-05 | 1.80e-03 ± 3.24e-04 | 6.11e-03 ± 4.05e-03 | 3.28e-05 ± 2.63e-05 | 5.19e-03 ± 1.77e-03 |
| **EDI Rainy and Dry Season** | Babies | Males | 2.30e-04 ± 8.09e-05 | 1.42e-05 ± 2.02e-05 | 3.22e-04 ± 1.72e-04 | 1.54e-02 ± 1.97e-02 | 5.38e-04 ± 1.33e-03 | 3.31e-06 ± 1.67e-06 | 8.22e-05 ± 1.83e-04 | 2.47e-05 ± 3.39e-05 | 3.18e-03 ± 5.65e-04 | 1.01e-02 ± 5.63e-03 | 5.90e-05 ± 3.63e-05 | 8.49e-03 ± 3.14e-03 |
|  |  | Females | 2.53e-04 ± 8.89e-05 | 1.56e-05 ± 2.23e-05 | 3.55e-04 ± 1.90e-04 | 1.69e-02 ± 2.17e-02 | 5.91e-04 ± 1.46e-03 | 3.64e-06 ± 1.83e-06 | 9.04e-05 ± 2.02e-04 | 2.72e-05 ± 3.73e-05 | 3.49e-03 ± 6.21e-04 | 1.11e-02 ± 6.19e-03 | 6.49e-05 ± 3.99e-05 | 9.34e-03 ± 3.46e-03 |
|  | Children | Males | 1.76e-04 ± 6.18e-05 | 1.08e-05 ± 1.55e-05 | 2.46e-04 ± 1.32e-04 | 1.18e-02 ± 1.50e-02 | 4.11e-04 ± 1.02e-03 | 2.53e-06 ± 1.27e-06 | 6.28e-05 ± 1.40e-04 | 1.89e-05 ± 2.59e-05 | 2.43e-03 ± 4.31e-04 | 7.74e-03 ± 4.30e-03 | 4.51e-05 ± 2.77e-05 | 6.48e-03 ± 2.40e-03 |
|  |  | Females | 1.92e-04 ± 6.74e-05 | 1.18e-05 ± 1.69e-05 | 2.69e-04 ± 1.44e-04 | 1.28e-02 ± 1.64e-02 | 4.48e-04 ± 1.11e-03 | 2.76e-06 ± 1.39e-06 | 6.85e-05 ± 1.53e-04 | 2.06e-05 ± 2.83e-05 | 2.65e-03 ± 4.71e-04 | 8.45e-03 ± 4.69e-03 | 4.92e-05 ± 3.03e-05 | 7.07e-03 ± 2.62e-03 |
|  | Adults | Males | 1.21e-04 ± 4.24e-05 | 7.43e-06 ± 1.06e-05 | 1.69e-04 ± 9.03e-05 | 8.06e-03 ± 1.03e-02 | 2.82e-04 ± 6.97e-04 | 1.73e-06 ± 8.73e-07 | 4.31e-05 ± 9.61e-05 | 1.29e-05 ± 1.78e-05 | 1.66e-03 ± 2.96e-04 | 5.31e-03 ± 2.95e-03 | 3.09e-05 ± 1.90e-05 | 4.45e-03 ± 1.65e-03 |
|  |  | Females | 1.27e-04 ± 4.45e-05 | 7.80e-06 ± 1.11e-05 | 1.77e-04 ± 9.49e-05 | 8.46e-03 ± 1.08e-02 | 2.96e-04 ± 7.31e-04 | 1.82e-06 ± 9.17e-07 | 4.52e-05 ± 1.01e-04 | 1.36e-05 ± 1.87e-05 | 1.75e-03 ± 3.11e-04 | 5.57e-03 ± 3.10e-03 | 3.25e-05 ± 1.99e-05 | 4.67e-03 ± 1.73e-03 |
| * EFSA, (2006);FAO/ WHO, (1995);USEPA, (2000)- Cu: Babies (<1 mg kg⁻¹); Children (<3 mg kg⁻¹); Adults (<5 mg kg⁻¹); Zn: Babies (<7 mg kg⁻¹); Children (<22 mg kg⁻¹); Adults (<25 mg kg⁻¹) | | | | | | | | | | | | | | |

**Table S8:** Estimated weekly intake (EWI) calculations concerning *Oreochromis niloticus* consumption by adult, adolescent, child, and infant males and females during the dry, rainy, and mixed seasons.

|  | **Group** | **Sex** | **As** | **Co** | **Cu** | **Fe** | **Mn** | **Mo** | **Ni** | **Pb** | **Rb** | **Ti** | **V** | **Zn** |
| --- | --- | --- | --- | --- | --- | --- | --- | --- | --- | --- | --- | --- | --- | --- |
| **EWI Rainy Season** | Babyies | Males | 1.36e-03 ± 5.47e-04 | 5.31e-05 ± 2.03e-05 | 1.74e-03 ± 5.31e-04 | 6.70e-02 ± 1.69e-02 | 1.38e-03 ± 5.18e-04 | NA | 3.15e-04 ± 2.13e-04 | 1.49e-04 ± 1.57e-04 | 2.14e-02 ± 3.63e-03 | 6.23e-02 ± 9.00e-03 | 4.07e-04 ± 8.18e-05 | 5.10e-02 ± 1.85e-02 |
|  |  | Females | 1.50e-03 ± 6.02e-04 | 5.84e-05 ± 2.23e-05 | 1.91e-03 ± 5.84e-04 | 7.36e-02 ± 1.86e-02 | 1.51e-03 ± 5.70e-04 | NA | 3.47e-04 ± 2.34e-04 | 1.64e-04 ± 1.73e-04 | 2.35e-02 ± 3.99e-03 | 6.86e-02 ± 9.90e-03 | 4.48e-04 ± 9.00e-05 | 5.61e-02 ± 2.03e-02 |
|  | Children | Males | 1.04e-03 ± 4.18e-04 | 4.06e-05 ± 1.55e-05 | 1.33e-03 ± 4.06e-04 | 5.11e-02 ± 1.29e-02 | 1.05e-03 ± 3.95e-04 | NA | 2.41e-04 ± 1.63e-04 | 1.14e-04 ± 1.20e-04 | 1.63e-02 ± 2.77e-03 | 4.76e-02 ± 6.87e-03 | 3.11e-04 ± 6.25e-05 | 3.90e-02 ± 1.41e-02 |
|  |  | Females | 1.14e-03 ± 4.56e-04 | 4.43e-05 ± 1.69e-05 | 1.45e-03 ± 4.43e-04 | 5.58e-02 ± 1.41e-02 | 1.15e-03 ± 4.31e-04 | NA | 2.63e-04 ± 1.78e-04 | 1.25e-04 ± 1.31e-04 | 1.78e-02 ± 3.02e-03 | 5.19e-02 ± 7.50e-03 | 3.39e-04 ± 6.82e-05 | 4.25e-02 ± 1.54e-02 |
|  | Adults | Males | 7.13e-04 ± 2.87e-04 | 2.78e-05 ± 1.06e-05 | 9.10e-04 ± 2.78e-04 | 3.51e-02 ± 8.84e-03 | 7.20e-04 ± 2.71e-04 | NA | 1.65e-04 ± 1.12e-04 | 7.83e-05 ± 8.22e-05 | 1.12e-02 ± 1.90e-03 | 3.27e-02 ± 4.71e-03 | 2.13e-04 ± 4.29e-05 | 2.67e-02 ± 9.69e-03 |
|  |  | Females | 7.49e-04 ± 3.01e-04 | 2.92e-05 ± 1.12e-05 | 9.55e-04 ± 2.92e-04 | 3.68e-02 ± 9.28e-03 | 7.56e-04 ± 2.85e-04 | NA | 1.73e-04 ± 1.17e-04 | 8.22e-05 ± 8.63e-05 | 1.17e-02 ± 1.99e-03 | 3.43e-02 ± 4.95e-03 | 2.24e-04 ± 4.50e-05 | 2.81e-02 ± 1.02e-02 |
| **EWI Dry Season** | Babies | Males | 1.81e-03 ± 5.06e-04 | 1.36e-04 ± 1.82e-04 | 2.67e-03 ± 1.43e-03 | 1.40e-01 ± 1.79e-01 | 5.66e-03 ± 1.22e-02 | 2.32e-05 ± 1.17e-05 | 7.82e-04 ± 1.69e-03 | 1.93e-04 ± 2.91e-04 | 2.29e-02 ± 4.12e-03 | 7.78e-02 ± 5.15e-02 | 4.18e-04 ± 3.35e-04 | 6.61e-02 ± 2.26e-02 |
|  |  | Females | 1.99e-03 ± 5.57e-04 | 1.50e-04 ± 2.00e-04 | 2.94e-03 ± 1.57e-03 | 1.54e-01 ± 1.97e-01 | 6.22e-03 ± 1.34e-02 | 2.55e-05 ± 1.28e-05 | 8.60e-04 ± 1.86e-03 | 2.12e-04 ± 3.20e-04 | 2.52e-02 ± 4.54e-03 | 8.55e-02 ± 5.67e-02 | 4.60e-04 ± 3.69e-04 | 7.27e-02 ± 2.48e-02 |
|  | Children | Males | 1.38e-03 ± 3.87e-04 | 1.04e-04 ± 1.39e-04 | 2.04e-03 ± 1.09e-03 | 1.07e-01 ± 1.37e-01 | 4.32e-03 ± 9.33e-03 | 1.77e-05 ± 8.91e-06 | 5.97e-04 ± 1.29e-03 | 1.47e-04 ± 2.22e-04 | 1.75e-02 ± 3.15e-03 | 5.94e-02 ± 3.94e-02 | 3.19e-04 ± 2.56e-04 | 5.05e-02 ± 1.72e-02 |
|  |  | Females | 1.51e-03 ± 4.22e-04 | 1.13e-04 ± 1.52e-04 | 2.22e-03 ± 1.19e-03 | 1.17e-01 ± 1.49e-01 | 4.71e-03 ± 1.02e-02 | 1.93e-05 ± 9.72e-06 | 6.51e-04 ± 1.41e-03 | 1.61e-04 ± 2.42e-04 | 1.91e-02 ± 3.44e-03 | 6.48e-02 ± 4.29e-02 | 3.48e-04 ± 2.79e-04 | 5.51e-02 ± 1.88e-02 |
|  | Adults | Males | 9.48e-04 ± 2.65e-04 | 7.12e-05 ± 9.53e-05 | 1.40e-03 ± 7.47e-04 | 7.33e-02 ± 9.36e-02 | 2.96e-03 ± 6.40e-03 | 1.21e-05 ± 6.11e-06 | 4.09e-04 ± 8.87e-04 | 1.01e-04 ± 1.52e-04 | 1.20e-02 ± 2.16e-03 | 4.07e-02 ± 2.70e-02 | 2.19e-04 ± 1.76e-04 | 3.46e-02 ± 1.18e-02 |
|  |  | Females | 9.96e-04 ± 2.78e-04 | 7.48e-05 ± 1.00e-04 | 1.47e-03 ± 7.85e-04 | 7.70e-02 ± 9.83e-02 | 3.11e-03 ± 6.72e-03 | 1.28e-05 ± 6.42e-06 | 4.30e-04 ± 9.31e-04 | 1.06e-04 ± 1.60e-04 | 1.26e-02 ± 2.27e-03 | 4.28e-02 ± 2.83e-02 | 2.30e-04 ± 1.84e-04 | 3.63e-02 ± 1.24e-02 |
| **EWI Rainy and Dry Season** | Babies | Males | 1.61e-03 ± 5.66e-04 | 9.93e-05 ± 1.42e-04 | 2.26e-03 ± 1.21e-03 | 1.08e-01 ± 1.38e-01 | 3.76e-03 ± 9.31e-03 | 2.32e-05 ± 1.17e-05 | 5.75e-04 ± 1.28e-03 | 1.73e-04 ± 2.37e-04 | 2.22e-02 ± 3.95e-03 | 7.09e-02 ± 3.94e-02 | 4.13e-04 ± 2.54e-04 | 5.94e-02 ± 2.20e-02 |
|  |  | Females | 1.77e-03 ± 6.23e-04 | 1.09e-04 ± 1.56e-04 | 2.48e-03 ± 1.33e-03 | 1.18e-01 ± 1.52e-01 | 4.14e-03 ± 1.02e-02 | 2.55e-05 ± 1.28e-05 | 6.33e-04 ± 1.41e-03 | 1.90e-04 ± 2.61e-04 | 2.44e-02 ± 4.35e-03 | 7.80e-02 ± 4.33e-02 | 4.55e-04 ± 2.80e-04 | 6.54e-02 ± 2.42e-02 |
|  | Children | Males | 1.23e-03 ± 4.32e-04 | 7.59e-05 ± 1.08e-04 | 1.72e-03 ± 9.22e-04 | 8.23e-02 ± 1.05e-01 | 2.87e-03 ± 7.11e-03 | 1.77e-05 ± 8.91e-06 | 4.40e-04 ± 9.81e-04 | 1.32e-04 ± 1.81e-04 | 1.70e-02 ± 3.02e-03 | 5.42e-02 ± 3.01e-02 | 3.16e-04 ± 1.94e-04 | 4.54e-02 ± 1.68e-02 |
|  |  | Females | 1.34e-03 ± 4.72e-04 | 8.28e-05 ± 1.18e-04 | 1.88e-03 ± 1.01e-03 | 8.97e-02 ± 1.15e-01 | 3.14e-03 ± 7.76e-03 | 1.93e-05 ± 9.72e-06 | 4.80e-04 ± 1.07e-03 | 1.44e-04 ± 1.98e-04 | 1.85e-02 ± 3.29e-03 | 5.91e-02 ± 3.28e-02 | 3.44e-04 ± 2.12e-04 | 4.95e-02 ± 1.83e-02 |
|  | Adults | Males | 8.44e-04 ± 2.96e-04 | 5.20e-05 ± 7.42e-05 | 1.18e-03 ± 6.32e-04 | 5.64e-02 ± 7.22e-02 | 1.97e-03 ± 4.88e-03 | 1.21e-05 ± 6.11e-06 | 3.01e-04 ± 6.73e-04 | 9.06e-05 ± 1.24e-04 | 1.16e-02 ± 2.07e-03 | 3.72e-02 ± 2.06e-02 | 2.16e-04 ± 1.33e-04 | 3.11e-02 ± 1.15e-02 |
|  |  | Females | 8.87e-04 ± 3.11e-04 | 5.46e-05 ± 7.79e-05 | 1.24e-03 ± 6.64e-04 | 5.92e-02 ± 7.58e-02 | 2.07e-03 ± 5.12e-03 | 1.28e-05 ± 6.42e-06 | 3.17e-04 ± 7.06e-04 | 9.51e-05 ± 1.31e-04 | 1.22e-02 ± 2.17e-03 | 3.90e-02 ± 2.17e-02 | 2.27e-04 ± 1.40e-04 | 3.27e-02 ± 1.21e-02 |
| * EFSA, (2006);FAO/ WHO, (1995);USEPA, (2000)- Cu: Baby (<7 mg kg^-1^); Child (<21 mg kg^-1^); Adult (<35 mg kg^-1^); Zn: Baby (<49 mg kg^-1^); Child (<91mg kg^-1^); Adult (<175 mg kg^-1^); | | | | | | | | | | | | | | |

**Table S9:** Target cancer risk (TCR) calculated for arsenic (As) from consumption of Oreochromis niloticus across age groups, consumption frequencies (including once per week), and dry, rainy, and mixed seasons. No values exceeded legal limits of 10^–6^ < TCR < 10^–4^ indicating acceptable risk and TCR > 10^–4^ indicating unacceptable risk (Antoine et al., 2017; USEPA, 2012).

| **Period** | **Group** | **Sex** | **As (1x)** | **As (2x)** | **As (3x)** | **As (4x)** | **As (5x)** |
| --- | --- | --- | --- | --- | --- | --- | --- |
| **TCR Dry Season** | Babies | Males | 7.37e-08 | 1.47e-07 | 2.21e-07 | 2.95e-07 | 3.68e-07 |
|  |  | Females | 8.10e-08 | 1.62e-07 | 2.43e-07 | 3.24e-07 | 4.05e-07 |
|  | Children | Males | 4.42e-08 | 8.84e-08 | 1.33e-07 | 1.77e-07 | 2.21e-07 |
|  |  | Females | 4.82e-08 | 9.65e-08 | 1.45e-07 | 1.93e-07 | 2.41e-07 |
|  | Adults | Males | 2.91e-08 | 5.83e-08 | 8.74e-08 | 1.17e-07 | 1.46e-07 |
|  |  | Females | 3.06e-08 | 6.12e-08 | 9.18e-08 | 1.22e-07 | 1.53e-07 |
| **TCR Rainy Season** | Babies | Males | 5.54e-08 | 1.11e-07 | 1.66e-07 | 2.22e-07 | 2.77e-07 |
|  |  | Females | 6.10e-08 | 1.22e-07 | 1.83e-07 | 2.44e-07 | 3.05e-07 |
|  | Children | Males | 3.33e-08 | 6.66e-08 | 9.98e-08 | 1.33e-07 | 1.66e-07 |
|  |  | Females | 3.63e-08 | 7.26e-08 | 1.09e-07 | 1.45e-07 | 1.82e-07 |
|  | Adults | Males | 2.19e-08 | 4.39e-08 | 6.58e-08 | 8.77e-08 | 1.10e-07 |
|  |  | Females | 2.30e-08 | 4.60e-08 | 6.91e-08 | 9.21e-08 | 1.15e-07 |
| **TCR Dry and Rainy Seasons** | Babies | Males | 6.56e-08 | 1.31e-07 | 1.97e-07 | 2.62e-07 | 3.28e-07 |
|  |  | Females | 7.22e-08 | 1.44e-07 | 2.17e-07 | 2.89e-07 | 3.61e-07 |
|  | Children | Males | 3.94e-08 | 7.88e-08 | 1.18e-07 | 1.58e-07 | 1.97e-07 |
|  |  | Females | 4.30e-08 | 8.59e-08 | 1.29e-07 | 1.72e-07 | 2.15e-07 |
|  | Adults | Males | 2.59e-08 | 5.19e-08 | 7.78e-08 | 1.04e-07 | 1.30e-07 |
|  |  | Females | 2.72e-08 | 5.45e-08 | 8.17e-08 | 1.09e-07 | 1.36e-07 |

**Table S10:** Target cancer risk (TCR) calculated for arsenic 1% (As) from consumption of *Oreochromis niloticus* across age groups, consumption frequencies (including once per week), and dry, rainy, and mixed seasons. No values exceeded legal limits of 10^–6^ < TCR < 10^–4^ indicating acceptable risk and TCR > 10^–4^ indicating unacceptable risk. (Antoine et al., 2017; USEPA, 2012).

| **Period** | **Group** | **Sex** | **As 1% (1x)** | **As 1% (2x)** | **As 1% (3x)** | **As 1% (4x)** | **As 1% (5x)** |
| --- | --- | --- | --- | --- | --- | --- | --- |
| **TCR Dry Season** | Babies | Males | 7.37e-10 | 1.47e-09 | 2.21e-09 | 2.95e-09 | 3.68e-09 |
|  |  | Females | 8.10e-10 | 1.62e-09 | 2.43e-09 | 3.24e-09 | 4.05e-09 |
|  | Children | Males | 4.42e-10 | 8.84e-10 | 1.33e-09 | 1.77e-09 | 2.21e-09 |
|  |  | Females | 4.82e-10 | 9.65e-10 | 1.45e-09 | 1.93e-09 | 2.41e-09 |
|  | Adults | Males | 2.91e-10 | 5.83e-10 | 8.74e-10 | 1.17e-09 | 1.46e-09 |
|  |  | Females | 3.06e-10 | 6.12e-10 | 9.18e-10 | 1.22e-09 | 1.53e-09 |
| **TCR Rainy Season** | Babies | Males | 5.54e-10 | 1.11e-09 | 1.66e-09 | 2.22e-09 | 2.77e-09 |
|  |  | Females | 6.10e-10 | 1.22e-09 | 1.83e-09 | 2.44e-09 | 3.05e-09 |
|  | Children | Males | 3.33e-10 | 6.66e-10 | 9.98e-10 | 1.33e-09 | 1.66e-09 |
|  |  | Females | 3.63e-10 | 7.26e-10 | 1.09e-09 | 1.45e-09 | 1.82e-09 |
|  | Adults | Males | 2.19e-10 | 4.39e-10 | 6.58e-10 | 8.77e-10 | 1.10e-09 |
|  |  | Females | 2.30e-10 | 4.60e-10 | 6.91e-10 | 9.21e-10 | 1.15e-09 |
| **TCR Dry and Rainy Seasons** | Babies | Males | 6.56e-10 | 1.31e-09 | 1.97e-09 | 2.62e-09 | 3.28e-09 |
|  |  | Females | 7.22e-10 | 1.44e-09 | 2.17e-09 | 2.89e-09 | 3.61e-09 |
|  | Children | Males | 3.94e-10 | 7.88e-10 | 1.18e-09 | 1.58e-09 | 1.97e-09 |
|  |  | Females | 4.30e-10 | 8.59e-10 | 1.29e-09 | 1.72e-09 | 2.15e-09 |
|  | Adults | Males | 2.59e-10 | 5.19e-10 | 7.78e-10 | 1.04e-09 | 1.30e-09 |
|  |  | Females | 2.72e-10 | 5.45e-10 | 8.17e-10 | 1.09e-09 | 1.36e-09 |

**Table S11:** Target cancer risk (TCR) calculated for arsenic 10% (As) from consumption of *Oreochromis niloticus* across age groups, consumption frequencies (including once per week), and dry, rainy, and mixed seasons. No values exceeded legal limits of 10^–6^ < TCR < 10^–4^ indicating acceptable risk and TCR > 10^–4^ indicating unacceptable risk. (Antoine et al., 2017; USEPA, 2012).

| **Period** | **Group** | **Sex** | **As 10% (1x)** | **As 10% (2x)** | **As 10% (3x)** | **As 10% (4x)** | **As 10% (5x)** |
| --- | --- | --- | --- | --- | --- | --- | --- |
| **TCR Dry Season** | Babies | Males | 7.37e-09 | 1.47e-08 | 2.21e-08 | 2.95e-08 | 3.68e-08 |
|  |  | Females | 8.10e-09 | 1.62e-08 | 2.43e-08 | 3.24e-08 | 4.05e-08 |
|  | Children | Males | 4.42e-09 | 8.84e-09 | 1.33e-08 | 1.77e-08 | 2.21e-08 |
|  |  | Females | 4.82e-09 | 9.65e-09 | 1.45e-08 | 1.93e-08 | 2.41e-08 |
|  | Adults | Males | 2.91e-09 | 5.83e-09 | 8.74e-09 | 1.17e-08 | 1.46e-08 |
|  |  | Females | 3.06e-09 | 6.12e-09 | 9.18e-09 | 1.22e-08 | 1.53e-08 |
| **TCR Rainy Season** | Babies | Males | 5.54e-09 | 1.11e-08 | 1.66e-08 | 2.22e-08 | 2.77e-08 |
|  |  | Females | 6.10e-09 | 1.22e-08 | 1.83e-08 | 2.44e-08 | 3.05e-08 |
|  | Children | Males | 3.33e-09 | 6.66e-09 | 9.98e-09 | 1.33e-08 | 1.66e-08 |
|  |  | Females | 3.63e-09 | 7.26e-09 | 1.09e-08 | 1.45e-08 | 1.82e-08 |
|  | Adults | Males | 2.19e-09 | 4.39e-09 | 6.58e-09 | 8.77e-09 | 1.10e-08 |
|  |  | Females | 2.30e-09 | 4.60e-09 | 6.91e-09 | 9.21e-09 | 1.15e-08 |
| **TCR Dry and Rainy Seasons** | Babies | Males | 6.56e-09 | 1.31e-08 | 1.97e-08 | 2.62e-08 | 3.28e-08 |
|  |  | Females | 7.22e-09 | 1.44e-08 | 2.17e-08 | 2.89e-08 | 3.61e-08 |
|  | Children | Males | 3.94e-09 | 7.88e-09 | 1.18e-08 | 1.58e-08 | 1.97e-08 |
|  |  | Females | 4.30e-09 | 8.59e-09 | 1.29e-08 | 1.72e-08 | 2.15e-08 |
|  | Adults | Males | 2.59e-09 | 5.19e-09 | 7.78e-09 | 1.04e-08 | 1.30e-08 |
|  |  | Females | 2.72e-09 | 5.45e-09 | 8.17e-09 | 1.09e-08 | 1.36e-08 |

**Table S12:** Target cancer risk (TCR) calculated for lead (Pb) from consumption of *Oreochromis niloticus* across age groups. consumption frequencies of 1-5 times per week and dry, rainy, and mixed seasons. No values exceeded legal limits of 10^–6^ < TCR < 10^–4^ indicating acceptable risk and TCR > 10^–4^ indicating unacceptable risk. (Antoine et al, 2017; USEPA, 2012).

| **Period** | **Group** | **Sex** | **Pb (1x)** | **Pb (2x)** | **Pb (3x)** | **Pb (4x)** | **Pb (5x)** |
| --- | --- | --- | --- | --- | --- | --- | --- |
| **TCR Dry Season** | Baby | Males | 4.45e-11 | 8.90e-11 | 1.34e-10 | 1.78e-10 | 2.23e-10 |
|  |  | Females | 4.90e-11 | 9.80e-11 | 1.47e-10 | 1.96e-10 | 2.45e-10 |
|  | Child | Males | 2.67e-11 | 5.34e-11 | 8.02e-11 | 1.07e-10 | 1.34e-10 |
|  |  | Females | 2.92e-11 | 5.83e-11 | 8.75e-11 | 1.17e-10 | 1.46e-10 |
|  | Adults | Males | 1.76e-11 | 3.52e-11 | 5.28e-11 | 7.04e-11 | 8.80e-11 |
|  |  | Females | 1.85e-11 | 3.70e-11 | 5.55e-11 | 7.40e-11 | 9.24e-11 |
| **TCR Rainy Season** | Baby | Males | 3.45e-11 | 6.89e-11 | 1.03e-10 | 1.38e-10 | 1.72e-10 |
|  |  | Females | 3.79e-11 | 7.58e-11 | 1.14e-10 | 1.52e-10 | 1.90e-10 |
|  | Child | Males | 2.07e-11 | 4.14e-11 | 6.21e-11 | 8.27e-11 | 1.03e-10 |
|  |  | Females | 2.26e-11 | 4.51e-11 | 6.77e-11 | 9.03e-11 | 1.13e-10 |
|  | Adults | Males | 1.36e-11 | 2.73e-11 | 4.09e-11 | 5.45e-11 | 6.81e-11 |
|  |  | Females | 1.43e-11 | 2.86e-11 | 4.29e-11 | 5.72e-11 | 7.16e-11 |
| **TCR Dry and Rainy Seasons** | Baby | Males | 3.99e-11 | 7.98e-11 | 1.20e-10 | 1.60e-10 | 1.99e-10 |
|  |  | Females | 4.39e-11 | 8.78e-11 | 1.32e-10 | 1.76e-10 | 2.19e-10 |
|  | Child | Males | 2.39e-11 | 4.79e-11 | 7.18e-11 | 9.58e-11 | 1.20e-10 |
|  |  | Females | 2.61e-11 | 5.22e-11 | 7.84e-11 | 1.04e-10 | 1.31e-10 |
|  | Adults | Males | 1.58e-11 | 3.16e-11 | 4.73e-11 | 6.31e-11 | 7.89e-11 |
|  |  | Females | 1.66e-11 | 3.31e-11 | 4.97e-11 | 6.63e-11 | 8.28e-11 |

**Table S13:** Target Hazard Quotient (THQ) values for arsenic (As), copper (Cu), nickel (Ni), vanadium (V) and zinc (Zn) as well as the Hazard Index (HI) calculated from the ingestion of *Oreochromis niloticus* one time a week by male and female infants, children, and adults on dry, rainy, and mixed seasons. No values exceeded legal limits of THQ < 1 indicating no carcinogenic effects expected and THQ > 1 indicating the possibility of adverse health effects (USEPA, 1989; USEPA, 2010).

| **Period** | **Group** | **Sex** | **As** | **As 1%** | **As 10%** | **Cu** | **Ni** | **V** | **Zn** | **HI** | **Frequency** |
| --- | --- | --- | --- | --- | --- | --- | --- | --- | --- | --- | --- |
| **THQ Dry Season** | Babies | Males | 1.64e-01 | 1.64e-03 | 1.64e-02 | 1.81e-02 | 1.89e-03 | 1.26e-03 | 5.98e-03 | 1.91e-01 | 1x |
|  |  | Females | 1.80e-01 | 1.80e-03 | 1.80e-02 | 1.99e-02 | 2.08e-03 | 1.39e-03 | 6.57e-03 | 2.10e-01 |  |
|  | Children | Males | 9.83e-02 | 9.83e-04 | 9.83e-03 | 1.09e-02 | 1.14e-03 | 7.56e-04 | 3.59e-03 | 1.15e-01 |  |
|  |  | Females | 1.07e-01 | 1.07e-03 | 1.07e-02 | 1.19e-02 | 1.24e-03 | 8.25e-04 | 3.91e-03 | 1.25e-01 |  |
|  | Adults | Males | 6.48e-02 | 6.48e-04 | 6.48e-03 | 7.16e-03 | 7.49e-04 | 4.98e-04 | 2.36e-03 | 7.55e-02 |  |
|  |  | Females | 6.80e-02 | 6.80e-04 | 6.80e-03 | 7.52e-03 | 7.87e-04 | 5.23e-04 | 2.48e-03 | 7.93e-02 |  |
| **THQ Rainy Season** | Babies | Males | 1.23e-01 | 1.23e-03 | 1.23e-02 | 1.18e-02 | 7.64e-04 | 1.23e-03 | 4.62e-03 | 1.42e-01 |  |
|  |  | Females | 1.36e-01 | 1.36e-03 | 1.36e-02 | 1.30e-02 | 8.41e-04 | 1.35e-03 | 5.08e-03 | 1.56e-01 |  |
|  | Children | Males | 7.39e-02 | 7.39e-04 | 7.39e-03 | 7.07e-03 | 4.59e-04 | 7.37e-04 | 2.77e-03 | 8.50e-02 |  |
|  |  | Females | 8.07e-02 | 8.07e-04 | 8.07e-03 | 7.72e-03 | 5.00e-04 | 8.04e-04 | 3.02e-03 | 9.27e-02 |  |
|  | Adults | Males | 4.87e-02 | 4.87e-04 | 4.87e-03 | 4.66e-03 | 3.02e-04 | 4.85e-04 | 1.83e-03 | 5.60e-02 |  |
|  |  | Females | 5.12e-02 | 5.12e-04 | 5.12e-03 | 4.89e-03 | 3.17e-04 | 5.10e-04 | 1.92e-03 | 5.88e-02 |  |
| **THQ Dry and Rainy Seasons** | Babies | Males | 1.46e-01 | 1.46e-01 | 1.46e-03 | 1.53e-02 | 1.39e-03 | 1.25e-03 | 5.37e-03 | 1.69e-01 |  |
|  |  | Females | 1.60e-01 | 1.60e-01 | 1.60e-03 | 1.68e-02 | 1.53e-03 | 1.37e-03 | 5.91e-03 | 1.86e-01 |  |
|  | Children | Males | 8.75e-02 | 8.75e-02 | 8.75e-04 | 9.19e-03 | 8.37e-04 | 7.48e-04 | 3.23e-03 | 1.02e-01 |  |
|  |  | Females | 9.55e-02 | 9.55e-02 | 9.55e-04 | 1.00e-02 | 9.13e-04 | 8.16e-04 | 3.52e-03 | 1.11e-01 |  |
|  | Adults | Males | 5.77e-02 | 5.77e-02 | 5.77e-04 | 6.05e-03 | 5.51e-04 | 4.93e-04 | 2.13e-03 | 6.69e-02 |  |
|  |  | Females | 6.05e-02 | 6.05e-02 | 6.05e-04 | 6.36e-03 | 5.79e-04 | 5.17e-04 | 2.23e-03 | 7.02e-02 |  |

**Table S14:** Target Hazard Quotient (THQ) values for arsenic (As), copper (Cu), nickel (Ni), vanadium (V) and zinc (Zn) as well as the Hazard Index (HI) calculated from the ingestion of *Oreochromis niloticus* two times a week by male and female infants, children, and adults on dry, rainy, and mixed seasons. No values exceeded legal limits of THQ < 1 indicating no carcinogenic effects expected and THQ > 1 indicating the possibility of adverse health effects (USEPA, 1989; USEPA, 2010).

| **Period** | **Group** | **Sex** | **As** | **As 1%** | **As 10%** | **Cu** | **Ni** | **V** | **Zn** | **HI** | **Frequency** |
| --- | --- | --- | --- | --- | --- | --- | --- | --- | --- | --- | --- |
| **THQ Dry Season** | Babies | Males | 3.27e-01 | 3.27e-01 | 3.27e-03 | 3.62e-02 | 3.79e-03 | 2.52e-03 | 1.20e-02 | 3.82e-01 | 2x |
|  |  | Females | 3.60e-01 | 3.60e-01 | 3.60e-03 | 3.98e-02 | 4.17e-03 | 2.77e-03 | 1.31e-02 | 4.20e-01 |  |
|  | Children | Males | 1.97e-01 | 1.97e-01 | 1.97e-03 | 2.17e-02 | 2.27e-03 | 1.51e-03 | 7.17e-03 | 2.29e-01 |  |
|  |  | Females | 2.14e-01 | 2.14e-01 | 2.14e-03 | 2.37e-02 | 2.48e-03 | 1.65e-03 | 7.83e-03 | 2.50e-01 |  |
|  | Adults | Males | 1.30e-01 | 1.30e-01 | 1.30e-03 | 1.43e-02 | 1.50e-03 | 9.97e-04 | 4.73e-03 | 1.51e-01 |  |
|  |  | Females | 1.36e-01 | 1.36e-01 | 1.36e-03 | 1.50e-02 | 1.57e-03 | 1.05e-03 | 4.96e-03 | 1.59e-01 |  |
| **THQ Rainy Season** | Babies | Males | 2.46e-01 | 2.46e-01 | 2.46e-03 | 2.36e-02 | 1.53e-03 | 2.46e-03 | 9.23e-03 | 2.82e-01 |  |
|  |  | Females | 2.71e-01 | 2.71e-01 | 2.71e-03 | 2.59e-02 | 1.68e-03 | 2.70e-03 | 1.02e-02 | 3.12e-01 |  |
|  | Children | Males | 1.48e-01 | 1.48e-01 | 1.48e-03 | 1.41e-02 | 9.17e-04 | 1.47e-03 | 5.54e-03 | 1.70e-01 |  |
|  |  | Females | 1.61e-01 | 1.61e-01 | 1.61e-03 | 1.54e-02 | 1.00e-03 | 1.61e-03 | 6.05e-03 | 1.85e-01 |  |
|  | Adults | Males | 9.75e-02 | 9.75e-02 | 9.75e-04 | 9.32e-03 | 6.04e-04 | 9.71e-04 | 3.65e-03 | 1.12e-01 |  |
|  |  | Females | 1.02e-01 | 1.02e-01 | 1.02e-03 | 9.79e-02 | 6.35e-04 | 1.02e-03 | 3.83e-03 | 1.18e-01 |  |
| **THQ Dry and Rainy Seasons** | Babies | Males | 2.92e-01 | 2.92e-01 | 2.92e-03 | 3.06e-02 | 2.79e-03 | 2.49e-03 | 1.07e-02 | 3.38e-01 |  |
|  |  | Females | 3.21e-01 | 3.21e-01 | 3.21e-03 | 3.37e-02 | 3.07e-03 | 2.74e-03 | 1.18e-02 | 3.72e-01 |  |
|  | Children | Males | 1.75e-01 | 1.75e-01 | 1.75e-03 | 1.84e-02 | 1.67e-03 | 1.50e-03 | 6.45e-03 | 2.03e-01 |  |
|  |  | Females | 1.91e-01 | 1.91e-01 | 1.91e-03 | 2.00e-02 | 1.83e-03 | 1.63e-03 | 7.04e-03 | 2.21e-01 |  |
|  | Adults | Males | 1.15e-01 | 1.15e-01 | 1.15e-03 | 1.21e-02 | 1.10e-03 | 9.85e-04 | 4.25e-03 | 1.34e-01 |  |
|  |  | Females | 1.21e-01 | 1.21e-01 | 1.21e-01 | 1.27e-03 | 1.16e-03 | 1.03e-03 | 4.46e-03 | 1.40e-01 |  |

**Table S15:** Target Hazard Quotient (THQ) values for arsenic (As), copper (Cu), nickel (Ni), vanadium (V) and zinc (Zn) as well as the Hazard Index (HI) calculated from the ingestion of *Oreochromis niloticus* three times a week by male and female infants, children. and adults during the dry, rainy, and grouped seasons. No values exceeded legal limits of THQ < 1 indicating no carcinogenic effects expected and THQ > 1 indicating the possibility of adverse health effects. (USEPA. 1989; USEPA. 2010).

| **Period** | **Group** | **Sex** | **As** | **As 1%** | **As 10%** | **Cu** | **Ni** | **V** | **Zn** | **HI** | **Frequency** |
| --- | --- | --- | --- | --- | --- | --- | --- | --- | --- | --- | --- |
| **THQ Dry Season** | Babies | Males | 4.91e-01 | 4.91e-01 | 4.91e-03 | 5.43e-02 | 5.68e-03 | 3.78e-03 | 1.79e-02 | 5.73e-01 | 3x |
|  |  | Females | 5.40e-01 | 5.40e-01 | 5.40e-03 | 5.97e-02 | 6.25e-03 | 4.16e-03 | 1.97e-02 | 6.30e-01 |  |
|  | Children | Males | 2.95e-01 | 2.95e-01 | 2.95e-03 | 3.26e-02 | 3.41e-03 | 2.27e-03 | 1.08e-02 | 3.44e-01 |  |
|  |  | Females | 3.22e-01 | 3.22e-01 | 3.22e-03 | 3.56e-02 | 3.72e-03 | 2.48e-03 | 1.17e-02 | 3.75e-01 |  |
|  | Adults | Males | 1.94e-01 | 1.94e-01 | 1.94e-03 | 2.15e-02 | 2.25e-03 | 1.50e-03 | 7.09e-03 | 2.27e-01 |  |
|  |  | Females | 2.04e-01 | 2.04e-01 | 2.04e-03 | 2.26e-02 | 2.36e-03 | 1.57e-03 | 7.45e-03 | 2.38e-01 |  |
| **THQ Rainy Season** | Babies | Males | 3.70e-01 | 3.70e-01 | 3.70e-03 | 3.53e-02 | 2.29e-03 | 3.68e-03 | 1.38e-02 | 4.25e-01 |  |
|  |  | Females | 4.07e-01 | 4.07e-01 | 4.07e-03 | 3.89e-02 | 2.52e-03 | 4.05e-03 | 1.52e-02 | 4.67e-01 |  |
|  | Children | Males | 2.22e-01 | 2.22e-01 | 2.22e-03 | 2.12e-02 | 1.38e-03 | 2.21e-03 | 8.31e-03 | 2.55e-01 |  |
|  |  | Females | 2.42e-01 | 2.42e-01 | 2.42e-03 | 2.31e-02 | 1.50e-03 | 2.41e-03 | 9.07e-03 | 2.78e-01 |  |
|  | Adults | Males | 1.46e-01 | 1.46e-01 | 1.46e-03 | 1.40e-02 | 9.07e-04 | 1.46e-03 | 5.48e-03 | 1.68e-01 |  |
|  |  | Females | 1.53e-01 | 1.53e-01 | 1.53e-03 | 1.47e-02 | 9.52e-04 | 1.53e-03 | 5.75e-03 | 1.76e-01 |  |
| **THQ Dry and Rainy Seasons** | Babies | Males | 4.37e-01 | 4.37e-01 | 4.37e-03 | 4.59e-02 | 4.18e-03 | 3.74e-03 | 1.61e-02 | 5.07e-01 |  |
|  |  | Females | 4.81e-01 | 4.81e-01 | 4.81e-03 | 5.05e-02 | 4.60e-03 | 4.11e-03 | 1.77e-02 | 5.58e-01 |  |
|  | Children | Males | 2.63e-01 | 2.63e-01 | 2.63e-03 | 2.76e-02 | 2.51e-03 | 2.24e-03 | 9.68e-03 | 3.05e-01 |  |
|  |  | Females | 2.86e-01 | 2.86e-01 | 2.86e-03 | 3.01e-02 | 2.74e-03 | 2.45e-03 | 1.06e-02 | 3.32e-01 |  |
|  | Adults | Males | 1.73e-01 | 1.73e-01 | 1.73e-03 | 1.82e-02 | 1.65e-03 | 1.48e-03 | 6.38e-03 | 2.01e-01 |  |
|  |  | Females | 1.82e-01 | 1.82e-01 | 1.82e-03 | 1.91e-02 | 1.74e-03 | 1.55e-03 | 6.70e-03 | 2.11e-01 |  |

**Table S16:** Target Hazard Quotient (THQ) values for arsenic (As), copper (Cu), nickel (Ni), vanadium (V) and zinc (Zn) as well as the Hazard Index (HI) calculated from the ingestion of *Oreochromis niloticus* four times a week by male and female infants, children. and adults during the dry, rainy, and grouped seasons. No values exceeded legal limits of THQ < 1 indicating no carcinogenic effects expected and THQ > 1 indicating the possibility of adverse health effects. (USEPA. 1989; USEPA. 2010).

| **Period** | **Group** | **Sex** | **As** | **As 1%** | **As 10%** | **Cu** | **Ni** | **V** | **Zn** | **HI** | **Frequency** |
| --- | --- | --- | --- | --- | --- | --- | --- | --- | --- | --- | --- |
| **THQ Dry Season** | Babies | Males | 6.55e-01 | 6.55e-01 | 6.55e-03 | 7.24e-02 | 7.58e-03 | 5.04e-03 | 2.39e-02 | 7.64e-01 | 4x |
|  |  | Females | 7.20e-01 | 7.20e-01 | 7.20e-03 | 7.97e-02 | 8.33e-03 | 5,55e-03 | 2,63e-02 | 8,40e-01 |  |
|  | Children | Males | 3.93e-01 | 3.93e-01 | 3.93e-03 | 4.35e-02 | 4.55e-03 | 3.03e-03 | 1.43e-02 | 4.58e-01 |  |
|  |  | Females | 4.29e-01 | 4.29e-01 | 4.29e-03 | 4.74e-02 | 4.96e-03 | 3.30e-03 | 1.57e-02 | 5.00e-01 |  |
|  | Adults | Males | 2.59e-01 | 2.59e-01 | 2.59e-03 | 2.86e-02 | 3.00e-03 | 1.99e-03 | 9.45e-03 | 3.02e-01 |  |
|  |  | Females | 2.72e-01 | 2.72e-01 | 2.72e-03 | 3.01e-02 | 3.15e-03 | 2.09e-03 | 9.93e-03 | 3.17e-01 |  |
| **THQ Rainy Season** | Babies | Males | 4.93e-01 | 4.93e-01 | 4.93e-03 | 4.71e-02 | 3.06e-03 | 4.91e-03 | 1.85e-02 | 5.66e-01 |  |
|  |  | Females | 5.42e-01 | 5.42e-01 | 5.42e-03 | 5.18e-02 | 3.36e-03 | 5.40e-03 | 2.03e-02 | 6.23e-01 |  |
|  | Children | Males | 2.96e-01 | 2.96e-01 | 2.96e-03 | 2.83e-02 | 1.83e-03 | 2.95e-03 | 1.11e-02 | 3.40e-01 |  |
|  |  | Females | 3.23e-01 | 3.23e-01 | 3.23e-03 | 3.09e-02 | 2.00e-03 | 3.22e-03 | 1.21e-02 | 3.71e-01 |  |
|  | Adults | Males | 1.95e-01 | 1.95e-01 | 1.95e-03 | 1.86e-02 | 1.21e-03 | 1.94e-03 | 7.30e-03 | 2.24e-01 |  |
|  |  | Females | 2.05e-01 | 2.05e-01 | 2.05e-03 | 1.96e-02 | 1.27e-03 | 2.04e-03 | 7.67e-03 | 2.35e-01 |  |
| **THQ Dry and Rainy Seasons** | Babies | Males | 5.83e-01 | 5.83e-01 | 5.83e-03 | 6.12e-02 | 5.58e-03 | 4.98e-03 | 2.15e-02 | 6.77e-01 |  |
|  |  | Females | 6.42e-01 | 6.42e-01 | 6.42e-03 | 6.74e-02 | 6.14e-03 | 5.48e-03 | 2.36e-02 | 7.44e-01 |  |
|  | Children | Males | 3.50e-01 | 3.50e-01 | 3.50e-03 | 3.68e-02 | 3.35e-03 | 2.99e-03 | 1.29e-02 | 4.06e-01 |  |
|  |  | Females | 3.82e-01 | 3.82e-01 | 3.82e-03 | 4.01e-02 | 3.65e-03 | 3.26e-03 | 1.41e-02 | 4.43e-01 |  |
|  | Adults | Males | 2.31e-01 | 2.31e-01 | 2.31e-03 | 2.42e-02 | 2.21e-03 | 1.97e-03 | 8.50e-03 | 2.68e-01 |  |
|  |  | Females | 2.42e-01 | 2.42e-01 | 2.42e-03 | 2.54e-02 | 2.32e-03 | 2.07e-03 | 8.93e-03 | 2.81e-01 |  |

**Table S17:** Target Target Hazard Quotient (THQ) values for arsenic (As), copper (Cu), nickel (Ni), vanadium (V) and zinc (Zn) as well as the Hazard Index (HI) calculated from the ingestion of *Oreochromis niloticus* five times a week by male and female infants, children. and adults during the dry, rainy, and grouped seasons. No values exceeded legal limits of THQ < 1 indicating no carcinogenic effects expected and THQ > 1 indicating the possibility of adverse health effects. (USEPA, 1989; USEPA, 2010).

| **Period** | **Group** | **Sex** | **As** | **As 1%** | **As 10%** | **Cu** | **Ni** | **V** | **Zn** | **HI** | **Frequency** |
| --- | --- | --- | --- | --- | --- | --- | --- | --- | --- | --- | --- |
| **THQ Dry Season** | Babies | Males | 8,19e-01 | 8,19e-01 | 8.19e-03 | 9.05e-02 | 9.47e-03 | 6.30e-03 | 2.99e-02 | 9.55e-01 | 5x |
|  |  | Females | 9.00e-01 | 9.00e-01 | 9.00e-03 | 9.96e-02 | 1.04e-02 | 6.93e-03 | 3.29e-02 | 1.05e+00 |  |
|  | Children | Males | 4.91e-01 | 4.91e-01 | 4.91e-03 | 5.43e-02 | 5.68e-03 | 3.78e-03 | 1.79e-02 | 5.73e-01 |  |
|  |  | Females | 5.36e-01 | 5.36e-01 | 5.36e-03 | 5.93e-02 | 6.20e-03 | 4.13e-03 | 1.96e-02 | 6.25e-01 |  |
|  | Adults | Males | 3.24e-01 | 3.24e-01 | 3.24e-03 | 3.58e-02 | 3.75e-03 | 2.49e-03 | 1.18e-02 | 3.78e-01 |  |
|  |  | Females | 3.40e-01 | 3.40e-01 | 3.40e-03 | 3.76e-02 | 3.93e-03 | 2.62e-03 | 1.24e-02 | 3.96e-01 |  |
| **THQ Rainy Season** | Babies | Males | 6.16e-01 | 6.16e-01 | 6.16e-03 | 5.89e-02 | 3.82e-03 | 6.14e-03 | 2.31e-02 | 7.08e-01 |  |
|  |  | Females | 6.78e-01 | 6.78e-01 | 6.78e-03 | 6.48e-02 | 4.20e-03 | 6.75e-03 | 2.54e-02 | 7.79e-01 |  |
|  | Children | Males | 3.70e-01 | 3.70e-01 | 3.70e-03 | 3.54e-02 | 2.29e-03 | 3.68e-03 | 1.39e-02 | 4.25e-01 |  |
|  |  | Females | 4.03e-01 | 4.03e-01 | 4.03e-03 | 3.86e-02 | 2.50e-03 | 4.02e-03 | 1.51e-02 | 4.64e-01 |  |
|  | Adults | Males | 2.44e-01 | 2.44e-01 | 2.44e-03 | 2.33e-02 | 1.51e-03 | 2.43e-03 | 9.13e-03 | 2.80e-01 |  |
|  |  | Females | 2.56e-01 | 2.56e-01 | 2.56e-03 | 2.45e-02 | 1.59e-03 | 2.55e-03 | 9.58e-03 | 2.94e-01 |  |
| **THQ Dry and Rainy Seasons** | Baby | Males | 7.29e-01 | 7.29e-01 | 7.29e-03 | 7.65e-02 | 6.97e-03 | 6.23e-03 | 2.69e-02 | 8.46e-01 |  |
|  |  | Females | 8.02e-01 | 8.02e-01 | 8.02e-03 | 8.42e-02 | 7.67e-03 | 6.85e-03 | 2.96e-02 | 9.30e-01 |  |
|  | Child | Males | 4.38e-01 | 4.38e-01 | 4.38e-03 | 4.59e-02 | 4.18e-03 | 3.74e-03 | 1.61e-02 | 5.08e-01 |  |
|  |  | Females | 4.77e-01 | 4.77e-01 | 4.77e-03 | 5.01e-02 | 4.56e-03 | 4.08e-03 | 1.76e-02 | 5.54e-01 |  |
|  | Adults | Males | 2.88e-01 | 2.88e-01 | 2.88e-03 | 3.03e-02 | 2.76e-03 | 2.46e-03 | 1.06e-02 | 3.34e-01 |  |
|  |  | Females | 3.03e-01 | 3.03e-01 | 3.03e-03 | 3.18e-02 | 2.90e-03 | 2.59e-03 | 1.12e-02 | 3,51e-01 |  |

**Table S18:** Significant Spearman correlation matrices between dissolved metals in surface water (suffix: _w) and total metal (suffix: _t) concentrations, during rainy season in Nile tilapia (Oreochromis niloticus) muscle samples. Only statistically significant correlations (p < 0.05) are shown.

| **Rainy**  **Season** | **As_w** | **Fe_w** | **Mg_w** | **Mn_w** | **Rb_w** | **V_w** | **Al_t** | **As_t** | **Co_t** | **Cu_t** | **Fe_t** | **Mn_t** | **Ni_t** | **Pb_t** | **Rb_t** | **Ti_t** | **V_t** | **Zn_t** |
| --- | --- | --- | --- | --- | --- | --- | --- | --- | --- | --- | --- | --- | --- | --- | --- | --- | --- | --- |
| As_w | 1.00 |  | 0.76 |  |  |  |  | -0.61 |  |  |  |  |  |  |  |  |  |  |
| Fe_w |  | 1.00 |  | -0.60 |  | -0.78 |  |  |  |  |  |  |  |  |  |  |  |  |
| Mg_w |  |  | 1.00 |  | -0.66 |  |  | -0.61 |  |  |  |  |  |  |  |  |  |  |
| Mn_w |  |  |  | 1.00 |  | 0.90 |  |  |  | -0.53 |  |  |  |  |  |  |  |  |
| Rb_w |  |  |  |  | 1.00 |  |  |  |  |  |  |  |  |  |  |  |  |  |
| V_w |  |  |  |  |  | 1.00 | 0.53 |  |  |  |  |  |  |  |  |  |  |  |
| Al_t |  |  |  |  |  |  | 1.00 |  |  |  |  |  |  |  |  |  |  |  |
| As_t |  |  |  |  |  |  |  | 1.00 |  |  |  |  |  |  |  |  |  |  |
| Co_t |  |  |  |  |  |  |  |  | 1.00 |  |  | 0.55 |  |  |  |  |  |  |
| Cu_t |  |  |  |  |  |  |  |  |  | 1.00 |  |  |  |  |  |  |  |  |
| Fe_t |  |  |  |  |  |  |  |  |  |  | 1.00 |  | 0.66 |  | -0.65 |  |  |  |
| Mn_t |  |  |  |  |  |  |  |  |  |  |  | 1.00 |  |  |  | -0.71 |  |  |
| Ni_t |  |  |  |  |  |  |  |  |  |  |  |  | 1.00 | -0.74 | -0.64 |  |  |  |
| Pb_t |  |  |  |  |  |  |  |  |  |  |  |  |  | 1.00 |  |  |  |  |
| Rb_t |  |  |  |  |  |  |  |  |  |  |  |  |  |  | 1.00 |  |  |  |
| Ti_t |  |  |  |  |  |  |  |  |  |  |  |  |  |  |  | 1.00 |  |  |
| V_t |  |  |  |  |  |  |  |  |  |  |  |  |  |  |  |  | 1.00 |  |
| Zn_t |  |  |  |  |  |  |  |  |  |  |  |  |  |  |  |  |  | 1.00 |

**Table S19:** Significant Spearman correlation matrices between dissolved metals in surface water (suffix: _w) and total metal (suffix: _t) concentrations, during dry season in Nile tilapia (Oreochromis niloticus) muscle samples. Only statistically significant correlations (p < 0.05) are shown.

| **Dry**  **Season** | **As_w** | **Fe_w** | **Mg_w** | **Mn_w** | **Rb_w** | **V_w** | **Al_t** | **As_t** | **Co_t** | **Cu_t** | **Fe_t** | **Mn_t** | **Ni_t** | **Pb_t** | **Rb_t** | **Ti_t** | **V_t** | **Zn_t** |
| --- | --- | --- | --- | --- | --- | --- | --- | --- | --- | --- | --- | --- | --- | --- | --- | --- | --- | --- |
| As_w | 1.00 |  | 0.75 |  |  |  |  |  |  |  |  |  |  | -0.80 |  |  |  |  |
| Fe_w |  | 1.00 |  |  |  | 0.52 |  |  |  |  |  |  |  |  |  |  |  |  |
| Mg_w |  |  | 1.00 |  | -0.60 |  |  |  |  |  |  |  |  | -0.57 |  |  |  |  |
| Mn_w |  |  |  | 1.00 |  | -0.59 |  |  |  |  |  |  |  |  |  |  |  |  |
| Rb_w |  |  |  |  | 1.00 | -0.53 |  |  |  |  |  |  |  |  |  |  |  |  |
| V_w |  |  |  |  |  | 1.00 |  | 0.65 |  | -0.55 |  |  |  |  |  |  |  |  |
| Al_t |  |  |  |  |  |  | 1.00 |  |  |  |  |  |  |  |  |  |  |  |
| As_t |  |  |  |  |  |  |  | 1.00 |  |  |  |  |  |  |  |  |  |  |
| Co_t |  |  |  |  |  |  |  |  | 1.00 |  |  |  |  |  |  |  |  |  |
| Cu_t |  |  |  |  |  |  |  |  |  | 1.00 |  |  |  |  |  |  |  |  |
| Fe_t |  |  |  |  |  |  |  |  |  |  | 1.00 |  |  |  |  |  |  |  |
| Mn_t |  |  |  |  |  |  |  |  |  |  |  | 1.00 |  |  |  |  |  |  |
| Ni_t |  |  |  |  |  |  |  |  |  |  |  |  | 1.00 |  |  |  | 0.52 |  |
| Pb_t |  |  |  |  |  |  |  |  |  |  |  |  |  | 1.00 |  |  |  |  |
| Rb_t |  |  |  |  |  |  |  |  |  |  |  |  |  |  | 1.00 |  | -0.76 | -0.56 |
| Ti_t |  |  |  |  |  |  |  |  |  |  |  |  |  |  |  | 1.00 |  |  |
| V_t |  |  |  |  |  |  |  |  |  |  |  |  |  |  |  |  | 1.00 |  |
| Zn_t |  |  |  |  |  |  |  |  |  |  |  |  |  |  |  |  |  | 1.00 |
